# Algorithm-Assisted Molecular Dynamics Simulations Revealed the Microscopic Mechanism by Which TX-100 and Biosurfactants Regulate the Separation of Heavy Oils from Solids

**DOI:** 10.3390/ma19143032

**Published:** 2026-07-14

**Authors:** Yutong Yang, Yuping Wang, Wu Wen, Jinze Du

**Affiliations:** 1 School of Telecommunications Engineering, Xidian University, Xi’an 710071, China; 2 School of Computer Science and Technology, Xidian University, Xi’an 710071, China; 3 Hainan Provincial Industrial Research Institute, Haikou 570203, China; 4 Zhejiang Institute of Tianjin University, Ningbo 315000, China

**Keywords:** molecular dynamics simulation, heavy oil, SARA fractions, surfactants, calcite interface, selective separation

## Abstract

**Highlights:**

**What are the main findings?**

- TX-100 screens SARA fractions by size via steric hindrance.
- Sophorolipid induces resin desorption via hydrogen bonding.
- Rhamnolipid drives aromatic desorption by electrostatic synergy.
- The C001 crystal face dominates adsorption in all systems.
- Mineral surfaces amplify kinetic differences among components.

**What are the implications of the main findings?**

- A molecular mechanism map guides surfactant selection.
- The C001 platform effect offers a universal separation strategy.
- The findings enable the rational design of selective surfactants.

**Abstract:**

To enhance quantitativeness and interpretability in identifying the mechanisms of complex oil–mineral–surfactant systems, this paper introduces an algorithmic molecular simulation analysis approach, transforming molecular dynamics trajectory data into comparable multidimensional molecular descriptors. Specifically, based on parameters such as radial distribution functions, mean square displacement, interface concentration distribution, adsorption energy attenuation, hydrogen bond statistics, and electrostatic interactions, an algorithm analysis framework was constructed covering “trajectory data acquisition—feature descriptor extraction—interface behavior recognition—separation mechanism classification.” This framework can identify differentiated regulatory patterns of different surfactants on SARA (saturates, aromatics, resins, asphaltenes) component migration, adsorption, and desorption behavior from a large amount of dynamic simulation data, thereby improving the structural expression and mechanism discrimination capabilities of molecular simulation results. In order to clarify the component-selective microscopic mechanisms of surfactants in the separation of heavy oil from oil sands, this work employs molecular dynamics simulations to study the interactions of the non-ionic surfactant TX-100 and the biosurfactants sophorolipid and rhamnolipid with the SARA fractions of heavy oil, both in the absence and presence of calcite mineral surfaces. The results show that all three surfactants act mainly through weak long-range interactions, but with distinct mechanisms: TX-100 preferentially screens small-molecule saturates through long-chain steric hindrance and hinders the diffusion of asphaltenes; sophorolipid promotes the preferential desorption of resins via hydrogen bonding; and rhamnolipid drives the desorption of aromatics at later stages through hydrophobic–electrostatic synergy. The C001 crystal surface exhibits the strongest adsorption affinity across all systems; the mineral surface overall prolongs the diffusion equilibrium time and amplifies the above kinetic differences. This study establishes three molecular-scale mechanisms—steric hindrance sieving, hydrogen-bond-promoted desorption, and electrostatically driven desorption—and reveals the universal adsorption platform effect of the C001 crystal surface, providing a theoretical basis for the molecular design of surfactants aimed at the selective separation of heavy oil components.

## 1. Introduction

With the diversification and in-depth development of the global energy structure, the economic exploitation of unconventional oil and gas resources has become a key link in ensuring energy security. Among them, oil sands are rich in reserves, but the traditional separation process of heavy oil, their core component, is inefficient, and both energy consumption and environmental pressure are high; this is due to heavy oil’s inherent high viscosity and strong interface adsorption with the mineral matrix [1,2]. Surfactant-assisted extraction technology is considered to be an effective way to solve this problem by regulating the interface properties.

Previous research has formed a broad consensus that the introduction of surfactants (such as anionic, non-ionic and biological surfactants) can effectively reduce the tension of the oil–solid interface and change the wettability of rocks, thus improving the recovery rate of heavy oil at a macro level [3,4,5,6,7,8]. In particular, surfactants with a unique molecular structure (such as double-tailed type and Gemini type) have been proven to be better than traditional single-stranded surfactants because of their stronger adsorption ability at the interface and lower critical micelle concentration [9,10,11,12]. This provides a direction for the selection of high-performance surfactants.

In recent years, in order to optimize the separation efficiency, researchers have carried out a lot of work from multiple angles. For example, Massarweh et al. [13] systematically reviewed the multiple macroscopic mechanisms of surfactant oil driving in improving the recovery rate (such as reducing interface tension and changing wettability) and discussed how to screen and optimize according to reservoir conditions and rock types, but the review is mainly based on laboratory and on-site scales. The performance evaluation did not reveal the specific difference in the role of key components of heavy oil such as surfactants and asphalt on a molecular scale. Wang et al. [14] focused on Gemini surfactants. By systematically changing the polyether chain length, interval base and hydrophobic alkyl chain length, they studied in detail the influence of structure on macro properties such as interface tension, wetting and emulsion stability, and established a clear structure–performance relationship; however, it is necessary to pay attention to the evolution of the macroscopic properties of fluids, and the work fails to clarify how these key structural advantages affect the competitive replacement behavior of surfactants on components such as colloid and asphalt in heavy oil at the molecular level. In addition, for biological surfactants, Li et al. [15] developed mouse plum glycolipid monoethanolamide (RL-MEA) with lower adsorption loss and higher oil-washing efficiency through chemical modification of mouse plum glycolipids and evaluated its critical micelle concentration and adsorption isotherm under high-temperature and high-salt conditions on a laboratory scale system. Most existing studies have emphasized surfactant synthesis, structural optimization, and macroscopic performance evaluation, including oil-washing efficiency. Nevertheless, the dynamic competitive adsorption and displacement mechanisms of modified surfactant molecules among heavy oil components and mineral surfaces have not been fully clarified. Liu et al. [16], for instance, applied dissipative particle dynamics to examine the competitive and cooperative adsorption of asphaltene and model surfactants (H_2_T_4_ and H_4_T_4_) at the oil–water interface. However, the composite model used in that study contained only asphaltene and did not capture the coexistence of saturates, aromatics, resins, and asphaltenes in real heavy oil. As a result, although the interaction of specific components was clarified, the findings are not sufficient to guide the selective separation or displacement of complex heavy oil systems. In general, existing studies have improved process optimization and macroscopic performance evaluation, but molecular-level issues such as preferential interactions, dynamic adsorption pathways, and quantitative structure–effect relationships between surfactants and multi-component heavy oil remain unclear.

Although a large number of macro experiments have confirmed that a variety of surfactants can improve the recovery rate of heavy oil, the existing research is mostly focused on process condition optimization and macro efficiency evaluation [17,18]. At present, there is still a lack of a systematic and clear mechanism explanation of how surfactants interact specifically with the molecular scale of heavy oil components, thereby differentiating and affecting the diffusion behavior, interface adsorption tendency and final separation path of each component. At the molecular level, it is still unclear which heavy oil components are preferentially associated with surfactant molecules, how these molecules bind to each other, and how competitive adsorption and displacement occur at the interface. The link between surfactant structure and the selective removal of specific oil components also remains insufficiently quantified. As a result, surfactant selection is still often based on empirical screening rather than clear molecular guidance. This limits the possibility of designing surfactants for specific heavy oil compositions and makes it difficult to build separation systems with both high efficiency and high selectivity.

From the perspective of computer simulation and algorithm analysis, the separation process of heavy oil is not a single physicochemical process but a complex dynamic system jointly determined by multi-component molecular structure, interfacial adsorption states, diffusion paths, interaction energies, and spatiotemporal distribution. Traditional experimental methods can evaluate surfactant performance based on macroscopic indicators such as recovery rate, contact angle, viscosity, and component content, but it is difficult to directly reveal differences in selective recognition, competitive adsorption, and migration pathways among different molecules at the microscopic level. Molecular dynamics simulation provides a computable research path for this problem. Further converting simulation trajectories into structured descriptors, algorithmically comparing and categorizing, and performing mechanism explanations can shift surfactant screening from empirical judgment to data-driven mechanistic identification.

In this sense, this paper does not merely use molecular dynamics software to obtain several simulated images but attempts to establish an algorithm-assisted analysis paradigm for the separation problem of heavy oil interfaces. This paradigm first generates multi-system trajectory data through molecular model construction and kinetic simulation, then extracts characteristic variables such as the RDF peak, MSD apparent diffusion coefficient, interfacial concentration peak, adsorption energy changes, hydrogen bond count, and electrostatic contribution, and finally identifies differentiated regulatory modes of TX-100, sophorolipid, and rhamnolipid for saturation, aroma, colloid, and asphaltene based on these variables. Thus, this paper can establish clear data mapping relationships between experimental phenomena and molecular mechanisms and also provides a methodological foundation for subsequent machine learning-based surfactant molecular screening, interface behavior prediction, and heavy oil separation system optimization.

Therefore, in order to explain the micromechanics of surfactants promoting heavy oil separation from the root cause, this study introduces an atomic-scale research tool—molecular dynamics simulation. We focus on three typical surfactants (non-ionic TX-100 and the biological surfactants locust sugar lipids and mouse plum sugar lipids) and build a multi-system composite model of four components of heavy oil SARA. Through systematic simulation and comparative analysis, it aims to quantitatively reveal the details of the interaction between different surfactants and various heavy oil components from multiple dimensions such as energy evolution, molecular diffusion, spatial distribution and interface adsorption. The goal of this study is to: (1) clarify the intensity, mode and essential differences between the interaction between different surfactants and various heavy oil components; (2) reveal the micro mechanism of diffusion kinetics and competitive adsorption behavior of each component in the interface area under the influence of surfactants; (3) establish the molecular structure of surfactants and the structural relationship between interface behavior, in addition to component separation selectivity providing theoretical basis and molecular design guidelines for the development of efficient green separation processes for complex heavy oil systems.

## 2. Materials and Methods

### 2.1. Materials

The oil sand sample was obtained from Buton Island, Indonesia. Its basic composition was determined as 0.06 wt% water, 28.84 wt% heavy oil, and 71.10 wt% solid minerals. All organic solvents, including toluene, o-xylene, p-xylene, m-xylene, n-heptane, n-pentane, cyclopentane, cyclohexane, and ethyl acetate, were of analytical grade supplied by Shanghai Aladdin Biochemical Technology Co., Ltd. (Shanghai, China). Three surfactants were studied: the non-ionic surfactant TX-100 (polyoxyethylene octylphenyl ether) and the biosurfactants sophorolipid and rhamnolipid were from Shanghai Aladdin Biochemical Technology Co., Ltd. (Shanghai, China). The heavy oil was separated into SARA fractions (saturates, aromatics, resins, and asphaltenes) using a standard method. The composition was found to be: saturates 18.12 wt%, aromatics 31.23 wt%, resins 28.32 wt%, and asphaltenes 22.33 wt%.

### 2.2. Experimental Procedures

#### 2.2.1. Solvent Extraction

The sample was first dried at 100 °C for 24 h. About 5.0 g of the dried sample was placed in a flask, and 30 mL of a mixed solvent was added. The solvent mixture contained toluene, xylene isomers, n-alkanes, and cyclic alkanes. Extraction was performed in a 25 °C water bath with stirring at 700 rpm for 60 min. The mixture was then transferred to a centrifuge tube and separated at 7000 rpm for 5 min. The upper liquid layer was collected. The solid residue was extracted three more times with fresh solvent until the extract was colorless. All oil-containing extracts were combined. The solvent was removed using a rotary evaporator at 80 °C under a vacuum of 0.098 MPa. The obtained heavy oil was further dried in a vacuum oven at 80 °C and 0.02 MPa for 6 h and weighed (m_1_). The total heavy oil content in the original sample (m_0_) was measured by Soxhlet extraction. The recovery rate was calculated as

Recovery (%) = (m_1_/m_0_) × 100 (1)

#### 2.2.2. SARA Fractionation

The extracted heavy oil was separated into four fractions (SARA) following a reported procedure [19]. Briefly, 1.0 g of heavy oil was mixed with 50 mL of n-heptane in a flask and treated with ultrasound at 50 °C for 30 min. After centrifugation (7000 rpm, 5 min), the supernatant was collected. The solid residue was extracted repeatedly with fresh n-heptane until the solvent was clear. The combined n-heptane solution was concentrated to about 5 mL. The remaining solid was dried under vacuum (80 °C, 0.08 MPa, 4 h) and weighed as asphaltenes.

Column chromatography was used to separate the other fractions. A column was packed with 150 g of activated neutral alumina mixed with n-heptane, topped with a layer of quartz sand. The concentrated n-heptane solution was added to the top. Sequential elution was performed with 100 mL of n-heptane (saturates), 100 mL of toluene (aromatics), and 100 mL of a toluene/methanol mixture (1:1 *v*/*v*; resins). Each fraction was concentrated and dried under vacuum (80 °C, 0.08 MPa, 4 h) before weighing.

#### 2.2.3. Basic Property Characterization

Elemental analysis (C, H) of the heavy oil was conducted using an elemental analyzer (Thermo Scientific FlashSmart, Bremen, Germany). The C/H atomic ratio was calculated. The viscosity of the extracted oil was measured at 25 °C using a rotational viscometer (Wells/Brookfield™ DV-III+, Brookfield Engineering Laboratories, Inc., Middleboro, MA, USA).

#### 2.2.4. Contact Angle Measurement

The wettability change of the solid surface after treatment was evaluated by contact angle measurement [20]. Dried solid particles were pressed into a smooth tablet. The contact angle of a deionized water droplet on the tablet surface was measured using an SL200B goniometer (Kino, Boston, MA, USA). A smaller contact angle indicates increased hydrophilicity.

### 2.3. Molecular Modeling Methods

#### 2.3.1. Model Construction

**Figure 1:**
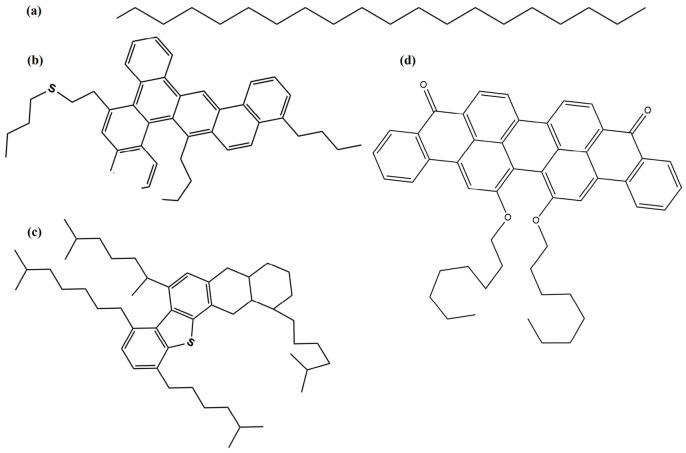
Molecular structure of SARA components: (**a**) saturates (C_20_H_42_), (**b**) aromatics (C_46_H_50_S), (**c**) resins (C_50_H_80_S), (**d**) asphaltenes (C_50_H_48_O_4_).

**Figure 2:**
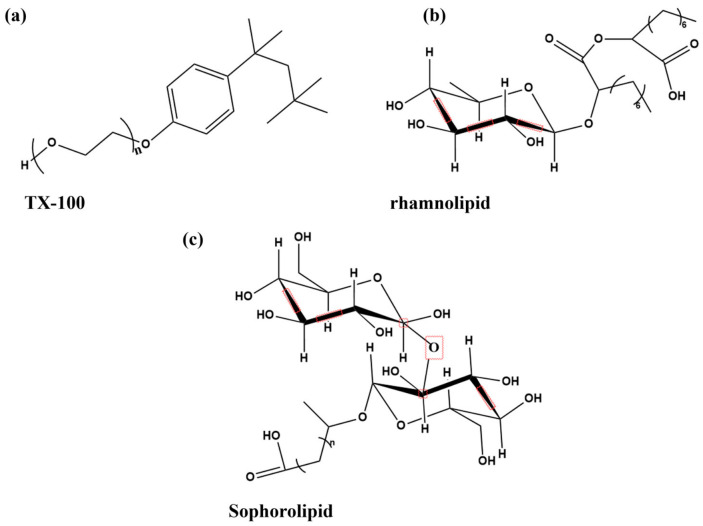
Molecular structures of the three surfactants used in this study: (**a**) TX-100, (**b**) rhamnolipid, and (**c**) sophorolipid (The red boxes were the active components).

Molecular dynamics (MD) simulations were conducted using BIOVIA Materials Studio 2020 (Version 20.1.0.2729). For the heavy oil, representative SARA molecules—including saturates, aromatics, resins, and asphaltenes—were selected. Four representative molecular models were built for the SARA fractions: a straight-chain alkane (C_22_H_46_) for saturates; a polyaromatic molecule (C_46_H_50_S, MW 635 g/mol) for aromatics [20,21,22,23]; a heteroatom-containing aromatic structure (C_50_H_80_S) for resins; and a large polar plane-like molecule (C_50_H_48_O_4_, MW 750 g/mol) for asphaltenes [24,25]. The SARA molecular models used in this study are simplified representative structures and cannot fully describe the complex molecular composition of real heavy oil fractions. The structures of the SARA fractions are shown Figure 1. Standard models were used for the surfactants (TX-100, Sophorolipid, Rhamnolipid). The molecular structures of the surfactants used in this study are shown in Figure 2.

#### 2.3.2. Construction of the Calcite Surface Model

The calcite (1 0 4) plane was adopted to model the carbonate mineral surface. The surface exposed Ca^2+^ and CO_3_^2−^ species. After energy minimization, the crystal was expanded into a supercell to provide a larger surface area. A 50 Å vacuum layer was introduced along the surface-normal direction to reduce interactions between periodic images. The final cell parameters were a = 72.86 Å, b = 29.94 Å, and c = 115.91 Å, with α = 90°, β = 90°, and γ = 120°. The CaCO_3_ stoichiometry was maintained, so the slab remained electrically neutral. The optimized slab was then used for the following adsorption simulations.

#### 2.3.3. Simulation Setup and Force Fields

##### Simulation Setup

This study considered two simulation systems: (i) first was the SARA component–surfactant system. The initial molecular configurations were generated using the Amorphous Cell module, followed by energy minimization in the Forcite module to obtain structurally stable models. This system was used to examine the diffusion behavior of the SARA components in the presence of surfactants. (ii) Second was the SARA component–surfactant–calcite system. A binary model consisting of SARA components and a calcite surface was first constructed using the Build Layers module to investigate the adsorption behavior of saturates, aromatics, resins, and asphaltenes on calcite, thereby representing the deposition of oil components on a realistic reservoir mineral surface. Subsequently, a three-layer model was established using the Build Layers module, in which the water–surfactant phase, SARA components, and calcite surface were arranged from top to bottom. This model was designed to simulate the washing process of oil sands by a surfactant solution. The simulation parameters for System 1 and System 2 are summarized in Table 1 and Table 2.

#### 2.3.4. Reproducibility and Statistical Analysis

##### Force Fields

The COMPASS force field has been widely used for condensed-phase organic molecules, hydrocarbons, surfactants, and organic–inorganic interfacial systems, and it can reasonably describe the van der Waals and electrostatic interactions involved in surfactant adsorption and oil–mineral interfacial behavior [26,27,28]. Long-range electrostatic interactions were treated using the Ewald summation method, with an accuracy of 10^−4^ kcal/mol. Van der Waals interactions were calculated by the atom-based summation method with a cutoff distance of 12.5 Å. The potential energy functions are expressed in Equation (2).

(2) $$E_{\mathrm{total}}=\sum_{\mathrm{bond}}E_{b}\left(b\right)+\sum_{\mathrm{angle}}E_{\theta }\left(\theta \right)+\sum_{\mathrm{dihedral}}E_{\emptyset }\left(\emptyset \right)+\sum_{\mathrm{cross}}E_{b}\left(b,\theta ,\emptyset \right)+\sum_{\mathrm{out}\text{−}\mathrm{of}\text{−}\mathrm{plane}}E_{X}\left(X\right)+E_{\mathrm{ele}}+E_{\mathrm{vdw}}$$

In the above equation, the first five terms correspond to bonded interactions, including bond stretching, angle bending, dihedral torsion, cross terms, and out-of-plane potential functions. Among them, b denotes the bond length between two covalently bonded atoms; θ represents the bond angle formed by three consecutively bonded atoms; ϕ is the dihedral angle (torsion) defined by a sequence of four atoms; and χ signifies the out-of-plane (inversion) coordinate, describing the displacement of an atom from the plane formed by three neighboring atoms. The cross terms account for the dynamic coupling between different internal coordinates. The last two terms represent non-bonded interactions, namely van der Waals and electrostatic interactions. The electrostatic and van der Waals interactions were calculated using the Ewald summation method and the Lennard-Jones 9-6 potential function, respectively, as expressed in Equations (3) and (4).

(3) $$E_{\mathrm{ele}}=\sum_{i>j}\frac{q_{i}q_{j}}{r_{\mathrm{ij}}}$$

(4) $$E_{\mathrm{vdw}}=\sum \varepsilon_{\mathrm{ij}}\left[2{\left(\frac{r_{\mathrm{ij}}^{0}}{r_{\mathrm{ij}}}\right)}^{9}-3{\left(\frac{r_{\mathrm{ij}}^{0}}{r_{\mathrm{ij}}}\right)}^{6}\right]$$

where i and j denote different atoms, q represents the atomic charge, r is the interatomic distance, and ε denotes the potential well depth. After completing Systems 1 and 2, MD simulations were conducted using the Forecite module under the NVT ensemble. the COMPASS II force field and the Smart algorithm were employed, with convergence criteria set as follows: an energy change of less than 0.001 kcal/mol and a force of less than 0.5 kcal/mol/Å. The system temperature was maintained at 300 K. A time step of 1 fs was employed, and the total simulation time was 2500 ps (System 1) and 4000 ps (System 2). Temperature was controlled using the Berendsen method.

In addition to RDF, concentration distribution, and MSD analyses, this study further calculates supplementary simulation descriptors to avoid overinterpretation of the molecular mechanisms based on a single descriptor alone. These descriptors include non-bonded interaction energy decomposition, hydrogen bond statistics, electrostatic descriptors, density redistribution at the calcite interface, adsorption energy attenuation, and steric accessibility parameters. See Tables S1–S4 for detailed results.

This study evaluated the repeatability and statistical reliability of the molecular dynamics simulation results by conducting three independent simulations for each system. The three simulations used the same force field, ensemble, temperature, time step, and total simulation time, but different initial velocity distributions were set. After the system reached equilibrium, the production-stage trajectories from the three independent simulations were analyzed separately.

For the analysis of the radial distribution function (RDF), concentration distribution, and mean square displacement (MSD), the results obtained from the three independent trajectories were first averaged, and the average was taken as the final curve; the fluctuations among the three simulations were expressed as standard deviations. For curve-based data, the standard deviation was calculated separately at the corresponding distance, spatial position, or simulation time point.

The apparent diffusion coefficient was calculated according to the quasi-linear region of the MSD curve in each independent simulation, and the final result was expressed as the average ± standard deviation of the three independent simulations. The above statistical processing was used to improve the reliability of the comparison between different surfactant systems and avoid drawing direct conclusions based on a single trajectory.

### 2.4. Interfacial Characterization

#### 2.4.1. Oil–Solid Interaction Force Measurement

The adhesion force between oil and mineral surfaces was measured using atomic force microscopy (AFM, Bruker Multimode 8, Bruker Corporation, Berlin, Germany) [26,27]. An AFM probe tip was modified with an asphalt-coated SiO_2_ microsphere. A calcite plate was used as the substrate. Force–distance curves were recorded in three different media: deionized water, 1 mM surfactant solution, and pure surfactant. The probe approach/retraction speed was set to 2 µm/s. Measurements were taken at over 100 different points for each sample to obtain an average adhesion force, which indicates how easily oil detaches from the surface [28].

#### 2.4.2. Zeta Potential Measurement

The surface charge of asphalt droplets and CaCO_3_ particles in surfactant solutions was characterized by measuring their zeta potential. A Zetasizer Nano ZS instrument (Malvern Instruments, Malvern, UK) was used. About 0.5 g of asphalt or CaCO_3_ powder was dispersed in 200 mL of surfactant solution, sonicated for 30 min, and then measured immediately. Each measurement was repeated three times.

### 2.5. Surfactant Adsorption Study

Batch adsorption experiments were conducted to measure how much surfactant adsorbed onto calcite powder. A total of 3.0 g of calcite powder was mixed with 30 mL of surfactant solution at different concentrations. The mixtures were shaken at 25 °C for 24 h. After centrifugation, the supernatant was collected. The surfactant concentration before and after adsorption was determined using UV–Vis spectroscopy. The adsorption amount was calculated from the concentration difference, and an adsorption isotherm was plotted. For the adsorption kinetics study, a solution with an initial concentration of 14,000 ppm was used. Samples were taken at different time intervals to analyze how adsorption changed over time [29].

## 3. Results

### 3.1. Overview of Simulation Systems and Equilibrium Validation

This section systematically studies the micro-interaction between the non-ionic surfactant TX 100 and two bio-source surfactants (locust glycolipids and mouse plum glycolipids) and the four components of heavy oil (saturated content, aromatic content, colloid, and asphalt) in mineral-free and mineral-containing systems. First of all, under the mineral-free system, the energy evolution, diffusion behavior, radial distribution and interface concentration distribution between the three surfactants and each component are examined one by one, and how the differences in molecular structure affect the interface adsorption and component dynamics are revealed. Secondly, after introducing the surface of calcite minerals, the regulatory effect of the mineral interface on the behavior of surfactants and its separation performance is clarified through adsorption experiments, zeta potential, atomic force microscopy adhesion measurement and molecular dynamics simulation. All simulation systems achieve energy and temperature balance within the set time scale. The relevant equilibrium evolution diagram is shown in the first part of the Supplementary Materials. This study aims to reveal the interaction mechanism between surfactant and heavy oil components, to reveal the difference in diffusion kinetics, interface adsorption selectivity and its intrinsic relationship with the molecular structure of surfactants at the atomic scale, and to provide a theoretical basis for the rational design of an efficient and highly selective heavy oil separation system.

### 3.2. Surfactant Behavior in Non-Mineral/Mineral Systems

#### 3.2.1. Energy Evolution and System Stability

After 100 ps of simulation, all systems reached a stable state in terms of both energy and temperature. Although the systems eventually became equilibrated, the energy distribution varied among different heavy oil components and surfactant systems. In the TX-100 system, the kinetic energy was more dominant for the saturated component, whereas the total potential energy made the major contribution in the aromatic, resin, and asphaltene systems. For the two biosurfactant systems, namely sophorolipid and rhamnolipid, the energy distribution showed a different pattern, especially in the aromatic and asphaltene systems, where the potential energy contribution was more pronounced. These differences suggest that the interaction strength and interaction mode between heavy oil components and surfactant molecules were not the same in different systems. In addition, the temperature remained stable throughout the simulation, fluctuating mainly within 288–309 K and showing only a slight deviation from the preset temperature of 298 K. No obvious temperature drift was observed, indicating that the simulations were consistent with the isothermal ensemble. More specifically, equilibrium was achieved after 100 ps, with negligible energy variation and temperature fluctuations maintained within 288–309 K.

#### 3.2.2. Radial Distribution Function (RDF) Analysis

**Figure 3:**
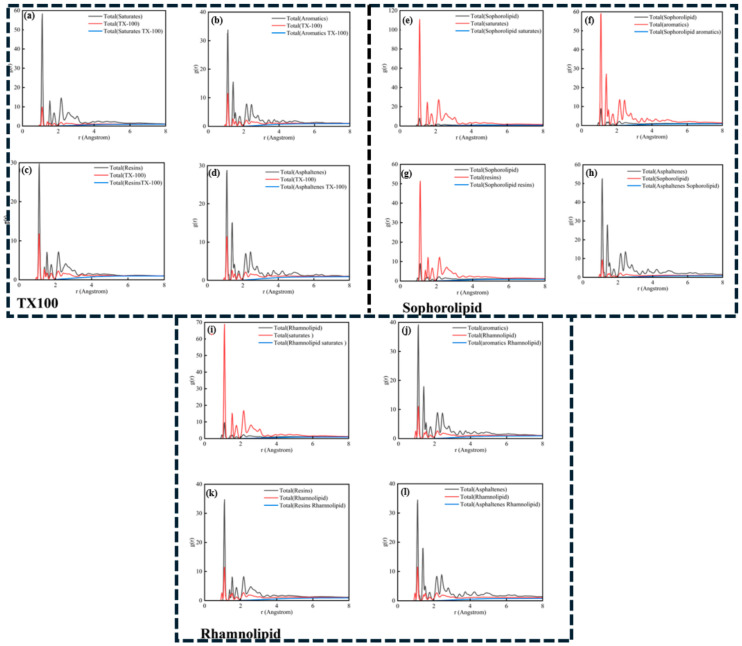
RDF between surfactants and SARA fractions in non-mineral systems: **TX-100-SARA system** (**a**) TX-100-saturates, (**b**) TX-100-aromatics, (**c**) TX-100-resins, (**d**) TX-100-asphaltenes; **Sophorolipid-SARA system** (**e**) Sophorolipid-saturates, (**f**) Sophorolipid-aromatics, (**g**) Sophorolipid-resins, (**h**) Sophorolipid-asphaltenes; **Rhamnolipid-SARA system** (**i**) Rhamnolipid-saturates, (**j**) Rhamnolipid-aromatics, (**k**) Rhamnolipid-resins, (**l**) Rhamnolipid-asphaltenes.

As shown in Figure 3, there are three main peaks in the RDF curves of the SARA fraction at about 1.11 Å, 1.41 Å and 2.17 Å, respectively. The RDF peak values are shown in Table 3. These multiple peaks reveal the specific accumulation patterns within or between component molecules. In stark contrast, there is only a main peak observed for TX-100, locust glycolipids and rat plum glycolipids at about 1.1 Å in all systems, which clearly shows that the intramolecular structure is relatively simple and the order is weak. More importantly, the RDF curve, which reflects the overall interaction between surfactants and component molecules, has no significant local correlation peaks in any of the 12 combination systems. In addition, when the atomic spacing is greater than about 6 Å (locust glycolipid–aromatic subsystem is about 5 Å), the RDF value of all systems is close to 1, achieving a uniform distribution of space. These consistent characteristics jointly confirm that although the molecular structure is very different, the interaction between the three surfactants and each heavy oil component is essentially a weak and long-range spatial association, rather than forming a strong and specific localized chemical bonding or coordination structure. This fundamental structural cognition lays the physical foundation for understanding the dynamic and reversible adsorption behavior of surfactants in the interface region and their subsequent differential regulation.

Figure S1 shows the RDF of the interaction between three surfactants and SARA components after the introduction of the calcite mineral surface. The RDF peak values are shown in Table S5. Consistent with the previous analysis results of non-mineral systems, the RDF curve of all heavy oil components still shows a short-range structural order that is richer than the surfactant itself, showing multiple characteristic peaks, while surfactants are still only ~1 in all systems. A main peak was observed at 1.1 Å, indicating that its intramolecular structure remains relatively simple. More importantly, there is also no obvious local correlation peak between the surfactant and any component in the mineral-containing systems, and except for the asphaltene system tending to balance after about 7 Å, the RDF value of the rest of the systems is close to 1 after the atomic spacing is greater than 6 Å. This once again confirms that the interaction between the three surfactants and heavy oil components is dominated by weak long-range spatial associations, rather than forming strong localized bonds, regardless of mineral interfaces.

Compared with non-mineral systems, the introduction of mineral surfaces does not change the essential type of interaction, but it may produce subtle modulation on the associated strength and spatial decay characteristics by providing additional adsorption sites (such as C001 crystal surfaces) and changing the local charge environment. This cross-system commonality further strengthens the following conclusion: the role of surfactants in the complex interface of oil–solid–water mainly depends on the physical space effect and polarity/electrostatic action determined by its molecular structure, rather than specific chemical bonding. This provides an important theoretical consistency for its application in the real oil sand separation environment.

#### 3.2.3. Concentration Distribution

**Figure 4:**
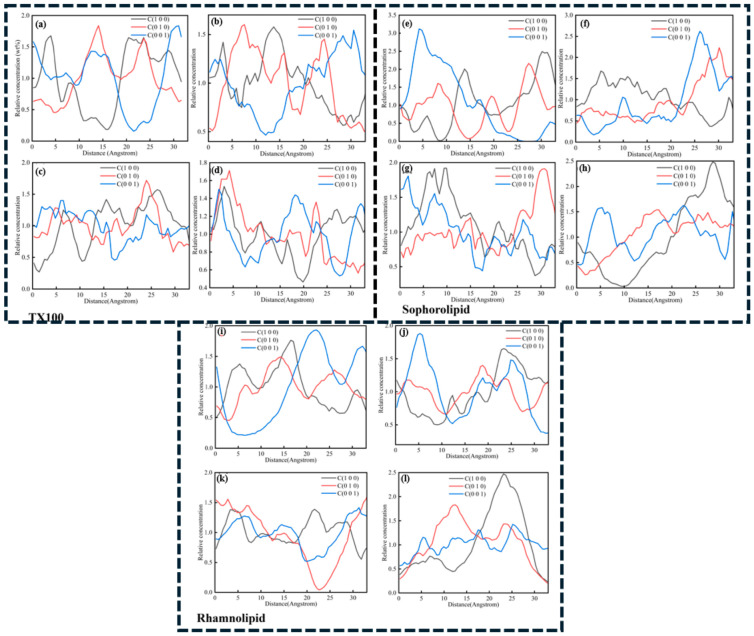
SARA concentration profiles of for different surfactants-SARA systems: **TX-100-SARA system** (**a**) TX-100-saturates, (**b**) TX-100-aromatics, (**c**) TX-100-resins, (**d**) TX-100-asphaltenes; **Sophorolipid-SARA system** (**e**) Sophorolipid-saturates, (**f**) Sophorolipid-aromatics, (**g**) Sophorolipid-resins, (**h**) Sophorolipid-asphaltenes; **Rhamnolipid-SARA system** (**i**) Rhamnolipid-saturates, (**j**) Rhamnolipid-aromatics, (**k**) Rhamnolipid-resins, (**l**) Rhamnolipid-asphaltenes (the curves represent the average values from three independent simulations).

Figure 4 shows the concentration distribution of the four SARA components along the x, y, and z directions in the presence of three different surfactants. In all systems, the concentration of each component exhibits obvious non-monotonic fluctuations, and the distribution differences among the three spatial directions are distinct. This indicates that the migration and accumulation of molecules in the system are dynamic and spatially selective processes dominated by molecular competition. In the TX-100 system, the concentration evolution of different components varies significantly along different directions. For example, along the z direction, the saturates show multi-stage fluctuations of “decrease–increase–decrease–increase”, while the aromatics generally follow a “decrease–increase–decrease” trend. The variation of resins is relatively mild, whereas the asphaltenes exhibit more complex “increase–decrease–increase–decrease–increase” oscillations. The relative order of concentration intensity among the three directions also changes with the spatial interval. For example, in the saturate system, the order changes from y direction > z direction > x direction in the 11–15 interval to x direction > y direction > z direction in the 19–24 interval. Similar interval-dependent rearrangements are also observed in the aromatics, resins, and asphaltene systems, reflecting a dynamic competitive balance. In the sophorolipid system, the concentration fluctuation along the z direction is the most complex. The saturates undergo a process of “rapid increase–continuous decrease–stabilization–rebound” along this direction, while the aromatics and asphaltenes show two main “increase–decrease” cycles. The resins first decrease, then increase, and finally decrease again. Although the x and y directions also present multi-stage fluctuations, their overall regularity is relatively weak. The relative order of directional accumulation also shows interval dependence. For example, in the saturate system, the order changes from z direction > y direction > x direction in the 2–11 interval to x direction > z direction > y direction in the 13–17 interval. Similar interval-specific rearrangements are also observed for the other components. In the rhamnolipid system, the concentration distribution again shows strong non-monotonicity and direction-dependent dynamics. Along the z direction, the saturates show “decrease–increase–decrease–increase” fluctuations, the aromatics generally increase first and then decrease, the resins show an overall upward trend, and the asphaltenes undergo a multi-stage “increase–decrease” cycle. The x and y directions show their own characteristic fluctuations, which are usually gentler than those along the z direction. The relative order of concentration intensity among the three directions also changes with the spatial interval. For example, for saturates, the order changes from x direction > y direction > z direction in the 3–10 interval to z direction > y direction > x direction in the 21–26 interval. Similar interval-dependent transformations are also observed in the aromatic, resin, and asphaltene systems.

**Figure 5:**
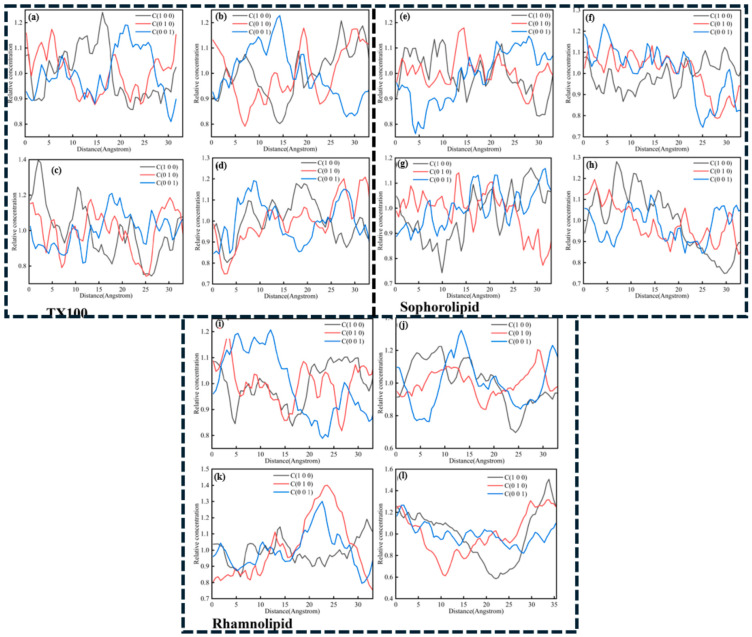
Surfactants concentration profiles of for different surfactants-SARA systems: **TX-100-SARA system** (**a**) TX-100-saturates, (**b**) TX-100-aromatics, (**c**) TX-100-resins, (**d**) TX-100-asphaltenes; **Sophorolipid-SARA system** (**e**) Sophorolipid-saturates, (**f**) Sophorolipid-aromatics, (**g**) Sophorolipid-resins, (**h**) Sophorolipid-asphaltenes; **Rhamnolipid-SARA system** (**i**) Rhamnolipid-saturates, (**j**) Rhamnolipid-aromatics, (**k**) Rhamnolipid-resins, (**l**) Rhamnolipid-asphaltenes (the curves represent the average values from three independent simulations).

Figure 5 depicts the concentration profiles of the three surfactants along the x, y, and z directions across the four distinct SARA components. A key finding is that the distribution of all surfactants is not static but exhibits significant non-monotonic dynamic fluctuations. More importantly, the distribution mode and relative concentration intensity along each direction are strongly and systematically regulated by the heavy oil component system, highlighting the obvious system dependence. TX-100 shows a recognizable but component-sensitive distribution behavior. Although its concentration fluctuates dynamically along each direction, in the key simulation intervals of most systems, for example, after about 18 ps in the saturate system, after about 12 ps in the aromatic system, and during 16–19 ps in the resin system, the z direction repeatedly shows final dominance or strong competitiveness. This indicates that, despite the initial dynamic competition, TX-100 still shows a potential preference for accumulation along the z direction in different molecular environments. In contrast, the biosurfactants sophorolipid and rhamnolipid show more complex and adaptive distribution kinetics. For sophorolipid, no single direction shows a general advantage in any of the component systems. Its dominant distribution direction switches dynamically according to the SARA environment. For example, the z direction dominates in the saturate system during 2–11 ps and in the aromatic system during 3–9 ps, while the x direction dominates at different stages of the saturate system during 13–17 ps, the aromatic system during 23–27 ps, and the asphaltene system during 9–13 ps. Rhamnolipid shows more significant dynamic reconstruction characteristics. Its concentration curve has clear stage-dependent features. For example, in the asphaltene system, the concentration along the x direction recovers only after a long period of continuous decline. Directional priority conversion also occurs within the same system, such as from the x direction > y direction > z direction to y direction > z direction > x direction in the aromatic system.

**Figure 6:**
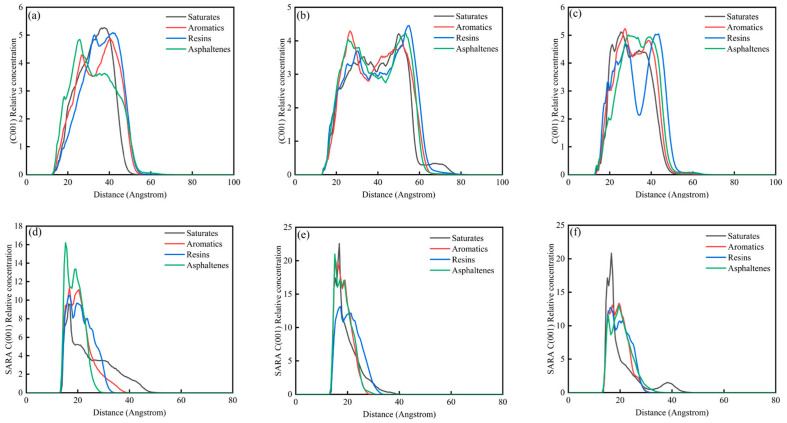
Interfacial concentration profiles in mineral systems: (**a**–**c**) TX-100, sophorolipid, and rhamnolipid relative concentration on crystal surfaces C001; (**d**–**f**) SARA fractions on crystal surfaces C001 (the curves represent the average values from three independent simulations).

Figure 6 shows the interfacial concentration distribution of each component of SARA/surfactants after the introduction of the calcite mineral surface. The calcite substrate exhibits a well-defined crystalline lattice structure with the surface exposed along the (001) crystallographic plane. The analysis of concentration profiles in this system was conducted exclusively along the C(001) direction, and the concentration of each component on this crystal surface is generally the strongest and reaches a peak in the range of about 15–20 ps. At the same time, the mineral environment has also strengthened the context dependence of different surfactant regulation modes: the adsorption peaks of each component in the sophorolipid system are more concentrated on the C001 crystal surface, while the asphaltenes in the rhamnolipid system show a unique “up-down-up” fluctuation and peak lag on the C001 crystal surface, reflecting the specific dynamics of its hydrophobic chain and complex molecules in the mineral-interface competition. These results show that the mineral surface does not change the basic framework of interfacial distribution but makes the adsorption-competition process more complex by introducing spatial heterogeneity and additional interaction sites and selectively amplifies the differentiated behavior of specific component–surfactant combinations, further emphasizing that efficient separation design must take into account the synergy between the molecular characteristics of surfactants, the properties of heavy oil components and the structure of mineral crystals. The adsorption behavior of TX-100 is relatively stable. The clear order dominated by the C001 crystal surface has been observed in different component systems. In summary, the introduction of mineral interfaces does not change the inherent preference of surfactants for the C001 crystal surface, but by introducing the spatial and chemical heterogeneity of the solid–liquid interface, the differences in competitive adsorption kinetics of different surfactant–component combinations are amplified. Especially for biosurfactants with specific functional groups (such as sugar rings and carboxyl groups of sophorolipid and anionic head groups and double chains of rhamnolipid), their adsorption behavior is more easily affected by the molecular properties of surrounding components, so as to show a dynamically adjusted interface distribution strategy for specific components under the universal adsorption framework. This further shows that in a complex oil–solid–water three-phase separation system, the efficiency of surfactants is the result of the synergy between its inherent molecular properties and the microenvironment of specific minerals and components.

#### 3.2.4. Dynamic Evolution of Diffusion

**Figure 7:**
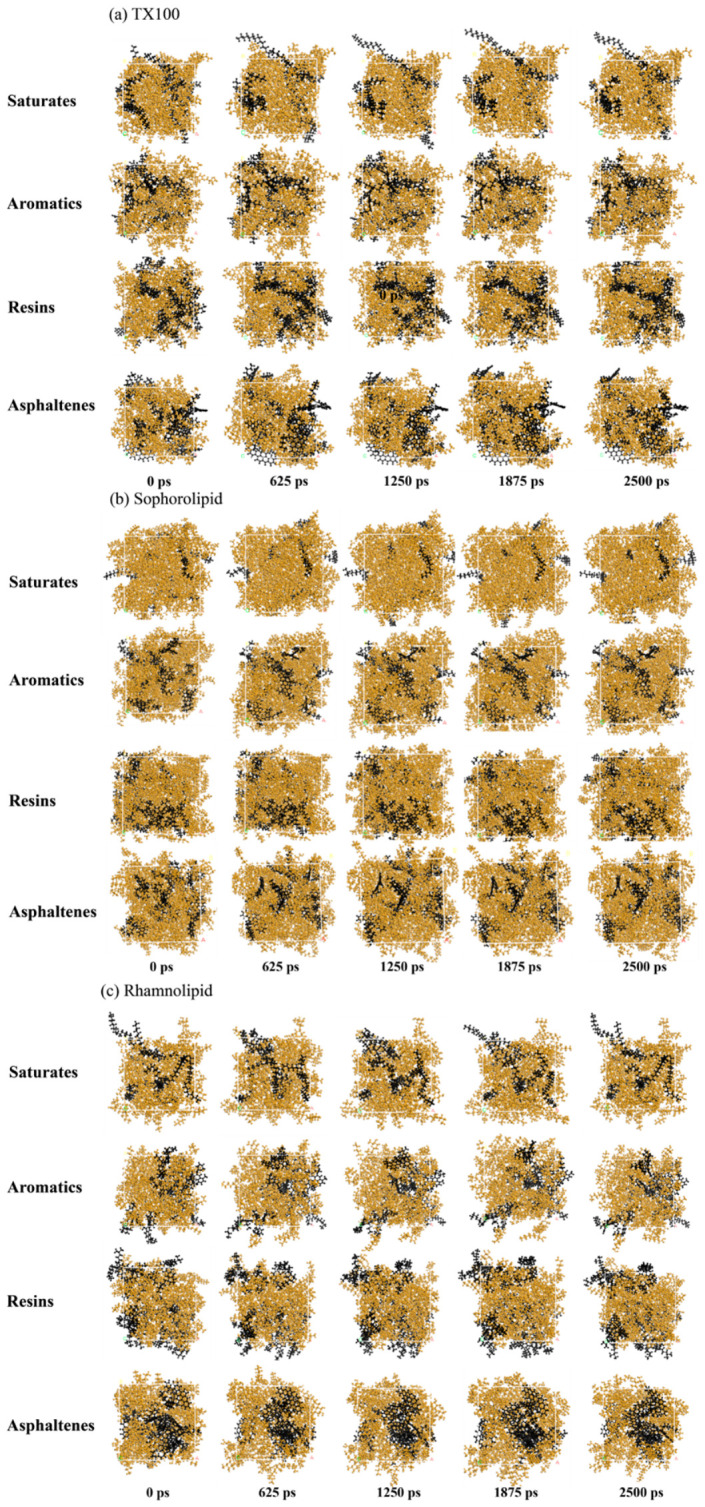
Snapshots from molecular dynamics simulations in non-mineral systems: (**a**) TX-100; (**b**) sophorolipid; (**c**) rhamnolipid. Yellow = surfactant and black = SARA fractions.

**Figure 8:**
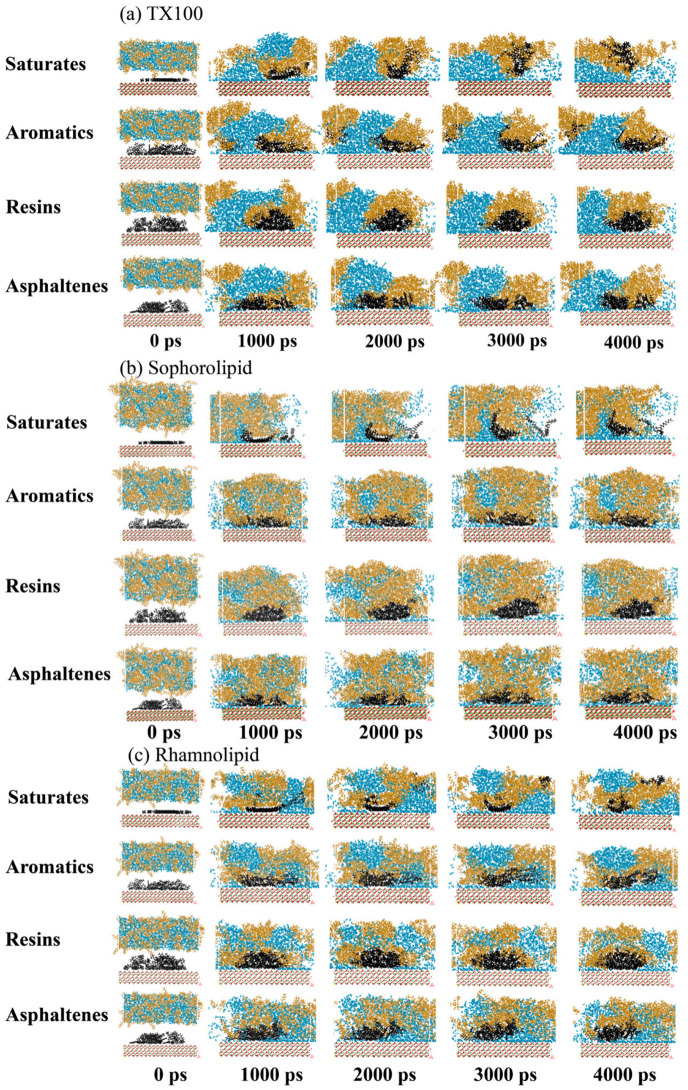
Snapshots from molecular dynamics simulations in mineral systems: (**a**) TX-100; (**b**) sophorolipid; (**c**) rhamnolipid. Blue = water, yellow = surfactant and black = SARA fractions.

Figure 7 presents molecular dynamics snapshots illustrating the structural evolution of the four SARA fractions in the presence of three surfactants. The images capture the transition of the system from an initially aggregated configuration to a progressively more dispersed state. Although a similar overall evolution trend is observed across all systems, marked differences appear in the spatial redistribution and interfacial behavior of the individual SARA fractions, which depend strongly on the type of surfactant involved. Figure 8 shows the diffusion conformation evolution process of SARA in the presence of three surfactants in a mineral system through molecular dynamics simulation snapshots. In the TX-100 system, aromatics and asphaltenes exhibit faster interfacial dispersion, while saturates and resins require a longer time. In the sophorolipid system, the order of diffusion kinetics changes significantly: saturates and resins show earlier dispersion, while aromatics and asphaltenes follow later. In the rhamnolipid system, resins disperse earlier, followed by aromatics, while saturates and asphaltenes are slower. Compared with the non-mineral system, the presence of the mineral surface generally prolongs the time required for each component to undergo structural evolution, indicating that the mineral interface acts as an additional adsorption site and slows down molecular migration to some extent. More importantly, the differential regulation of component diffusion behavior by different surfactants is still maintained in the mineral environment: TX-100 promotes relatively faster dispersion of aromatics and asphaltenes; sophorolipid preferentially accelerates the dispersion of saturates and resins; and rhamnolipid maintains its strong dispersion tendency toward the resin fraction. This demonstrates that although the mineral interface increases migration resistance, the molecular interaction mechanisms of surfactants remain the key factors governing differences in component diffusion behavior. The differences in these time scales provide an important molecular simulation basis for designing separation strategies under mineral-containing conditions.

#### 3.2.5. Mean Square Displacement (MSD) Analysis

**Figure 9:**
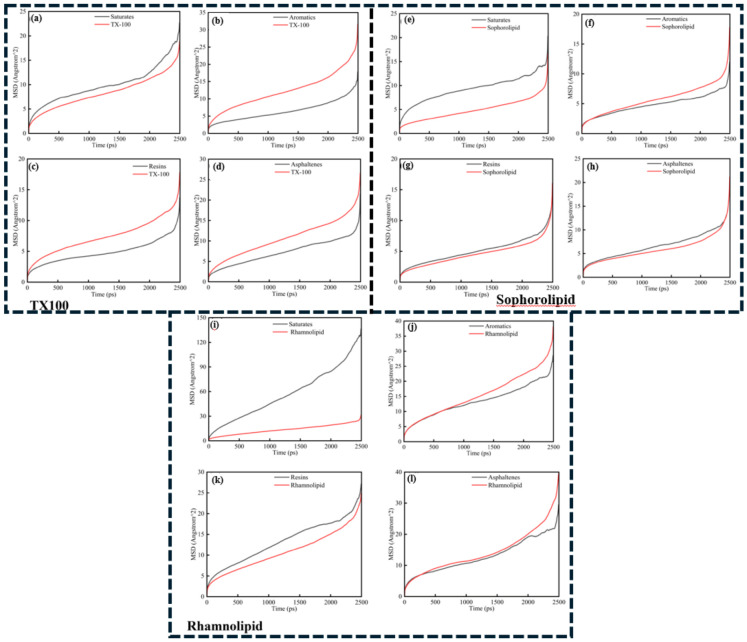
MSD curves for the SARA and TX-100, sophorolipid and rhamnolipid in non-mineral systems: **TX-100-SARA system** (**a**) TX-100-saturates, (**b**) TX-100-aromatics, (**c**) TX-100-resins, (**d**) TX-100-asphaltenes; **Sophorolipid-SARA system** (**e**) Sophorolipid-saturates, (**f**) Sophorolipid-aromatics, (**g**) Sophorolipid-resins, (**h**) Sophorolipid-asphaltenes; **Rhamnolipid-SARA system** (**i**) Rhamnolipid-saturates, (**j**) Rhamnolipid-aromatics, (**k**) Rhamnolipid-resins, (**l**) Rhamnolipid-asphaltenes (the curves represent the average values from three independent simulations).

This study uses the apparent diffusion coefficient ($D_{app}$) to characterize the migration behavior of surfactants and SARA components. Generally speaking, the larger the Dapp value, the stronger the surface migration ability of the corresponding molecule under the current simulation conditions. According to the Einstein relation, the diffusion coefficient can be calculated from the slope of the MSD curve, as shown in Equation (5).

(5) $$D=\frac{1}{6}\underset{t\to \infty }{\lim}\frac{d}{dt}{\left|r_{i}(t)-r_{i}(0)\right|}^{2}$$

where ${\left|r_{i}(t)-r_{i}(0)\right|}^{2}$ represents the MSD and r_i_(0) and r_i_(t) are the position vectors of particle i at the initial time and time t, respectively. It should be noted that the MSD curves obtained in this work do not show a single ideal linear regime over the entire simulation time but instead exhibit a staged evolution. This behavior is mainly related to the concentrated multi-component nature of the SARA–surfactant systems and, in the mineral-containing systems, to additional interfacial adsorption on calcite. The initial rapid increase in MSD can be attributed to local molecular relaxation and short-range rearrangement from the initial configurations, whereas the middle plateau-like region suggests transient confinement or caging of SARA molecules within oil aggregates, surfactant-associated domains, or calcite adsorption sites. The late-stage increase is likely associated with collective rearrangement, desorption, and enhanced dispersion promoted by surfactant molecules. Therefore, the S-shaped MSD profiles observed here reflect intermittent and interface-regulated diffusion rather than ideal homogeneous Brownian diffusion. For this reason, the diffusion coefficients listed in Table 4 should be regarded as apparent diffusion coefficients obtained from the quasi-linear regions of the MSD curves and are mainly used to compare the relative diffusion behavior of different SARA fractions and surfactant systems. We performed a linear regression on the MSD vs. time plot within the equilibrated period (from t_1_ to t_2_). Figure 9 presents MSD curves of the diffusion behavior of SARA and corresponding surfactants. The analysis shows that the MSD of all systems gradually increases over time at the beginning of the simulation, indicating that the system has not yet reached diffusion equilibrium. However, the relative relationship between the diffusion capacity of components and surfactants, as well as the time points for significant acceleration in the MSD curves, both show strong system dependence and component selectivity. The relative order of diffusion capacity varies across different surfactant systems. In the TX-100 system, the diffusion coefficient of saturates is higher than that of TX-100 itself, while the diffusion coefficients of aromatics, resins and asphaltenes are lower than TX-100. In the sophorolipid system, the diffusion coefficients of saturates, resins and asphaltenes are higher than that of sophorolipid throughout the simulation period, while the diffusion capacity of aromatics is weaker than that of sophorolipid after 150 ps. In the rhamnolipid system, the diffusion coefficient of saturates and resins is always higher than that of rhamnolipid, while the diffusion of aromatics and asphaltenes is surpassed by rhamnolipid in the later stage of simulation (after about 700 ps and 300 ps, respectively). The accelerated evolution characteristics of MSD further reveal the differences in diffusion dynamics. The MSD curve of each component shows obvious accelerated growth in the middle and late stages of the simulation, but their starting times are different. In the TX-100 system, the acceleration points for saturates, aromatics and resins/asphaltenes are around 1900 ps, 2000 ps and 2400 ps respectively, while the acceleration point of TX-100 itself is between 2300 and 2400 ps. In the sophorolipid and rhamnolipid systems, the MSD acceleration points of each component and the surfactant itself are also distributed in the 2000–2400 ps range, but the specific times vary depending on the component and system. For example, in the rhamnolipid system, the rapid growth of resins and asphaltenes begins at about 2200 ps and 2400 ps, respectively. The corresponding MSD diffusion coefficients are shown in Table 4.

**Figure 10:**
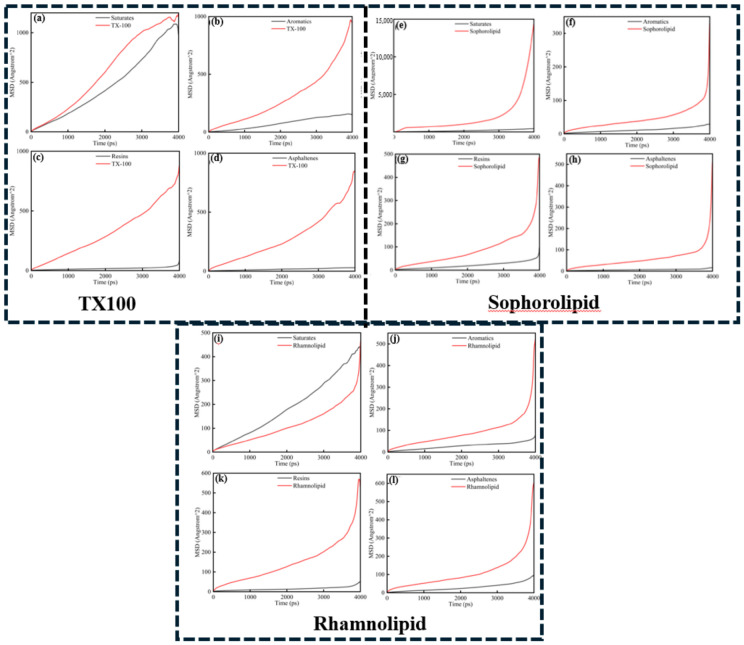
MSD curves for the SARA and TX-100, sophorolipid and rhamnolipid in mineral systems: **TX-100-SARA system** (**a**) TX-100-saturates, (**b**) TX-100-aromatics, (**c**) TX-100-resins, (**d**) TX-100-asphaltenes; **Sophorolipid-SARA system** (**e**) Sophorolipid-saturates, (**f**) Sophorolipid-aromatics, (**g**) Sophorolipid-resins, (**h**) Sophorolipid-asphaltenes; **Rhamnolipid-SARA system** (**i**) Rhamnolipid-saturates, (**j**) Rhamnolipid-aromatics, (**k**) Rhamnolipid-resins, (**l**) Rhamnolipid-asphaltenes (the curves represent the average values from three independent simulations).

Figure 10 shows MSD curves of the SARA and corresponding surfactants in the mineral systems. Compared with the non-mineral system, the most significant change is the general extension of the time to reach diffusion equilibrium. The MSD curves of all components maintained an upward trend over longer simulation times, and the starting points of their significant accelerated growth were also postponed accordingly. For example, in the TX-100 system, the rapid-rise points of each component are delayed to after 2300 ps; in the two biosurfactant systems, these time points are further delayed to beyond 3300 ps. This directly confirms that the mineral surface, as a strong adsorption site, increases the energy barrier for molecular diffusion and slows down its process of achieving dynamic equilibrium. Secondly, the order of the relative diffusion ability between surfactants and components has undergone characteristic changes. In the non-mineral TX-100 system, the diffusion coefficient of saturates was higher than that of TX-100; in the presence of minerals, the diffusion coefficient of TX-100 in all systems was higher than that of the corresponding heavy oil components. This reversal shows that there is a difference in the adsorption of surfactants and heavy oil components on the mineral surface: TX-100 may be more prone to dynamic adsorption–desorption on the mineral surface, thus showing faster apparent diffusion at the macro level, while heavy oil components may be limited by stronger interfacial-retention effects. For rhamnolipid, its “late-stage drive” effect (diffusion coefficient surpassing that of the components) on aromatic and asphaltene diffusion in mineral-containing systems still exists, but the time point of surpassing (after about 700 ps and 300 ps, respectively) occurs earlier than in non-mineral systems, suggesting that the mineral interface may strengthen the effectiveness of its electrostatic-repulsion effect. The corresponding MSD diffusion coefficients are shown in Table 5.

These quantitative results jointly reveal that in the complex interfacial region where oil, solid and water coexist, diffusion behavior is the result of the dynamic interplay among the surfactant molecular mechanism, heavy oil component properties and mineral-surface characteristics. The mineral environment not only increases the overall diffusion resistance, but more importantly, it selectively modulates the relative migration ability of different surfactant–component pairs. This provides key dynamic parameters and a theoretical basis that are closer to real-world conditions for utilizing diffusion-kinetics differences to achieve selective enhanced separation in actual oil sand separation processes.

#### 3.2.6. Integration of Available Experimental Characterization and Simulation Descriptors

In order to further strengthen the connection between experimental characterization results and molecular simulation analysis, this section comprehensively discusses the existing experimental information and the molecular descriptors obtained from simulation. The experimental results show that the heavy oil content in the oil sand sample used in this study is 28.84 wt% and the solid mineral content is 71.10 wt%, indicating that the organic components in the system coexist with mineral phases and have typical oil–solid composite interfacial characteristics. In addition, the results of the SARA fraction analysis show that the extracted heavy oil is composed of 18.12 wt% saturates, 31.23 wt% aromatics, 28.32 wt% resins, and 22.33 wt% asphaltenes. Rhamnolipid may facilitate the later-stage migration of aromatics through combined hydrophobic and electrostatic contributions. The above experimental results provide a compositional basis for the selection of representative SARA molecular models and the construction of a mineral-containing interfacial simulation system.

In terms of simulation analysis, RDF peak intensity can be used to characterize the local correlation between surfactants and different SARA components; concentration distribution can reflect the enrichment behavior and adsorption tendency of molecules in the interfacial region; and the apparent diffusion coefficient is used to compare the relative migration ability of different components in various surfactant systems. The experimental results, including heavy oil recovery, contact angle, the C/H ratio, SARA composition, zeta potential, AFM adhesion force, and adsorption kinetics, were further correlated with molecular simulation descriptors such as RDF peak intensity, concentration distribution, interfacial enrichment behavior, and apparent diffusion coefficients. Therefore, this article mainly analyzes the correspondence between the existing experimental characterization results and molecular simulation descriptors at this stage. Specifically, the high mineral content in the oil sand sample shows the necessity of introducing the calcite interface into the simulation system, and the SARA composition obtained in the experiment provides a basis for the establishment of a four-component heavy oil model. On this basis, the simulation results further reveal the regulatory effect of different surfactant structures on the interfacial distribution and diffusion behavior of the heavy oil components identified by these experiments.

#### 3.2.7. Molecular Structure Dictates Interfacial Role in Non-Mineral Systems

The molecular body of TX-100 is a long polyoxyethylene chain. The simulation results show that this structural feature makes it mainly play the role of a spatial regulator at the interface. Its long chain does not have a strong chemical effect with specific components but forms a dynamic “barrier” with a certain volume in the interface area. This barrier effect (spatial resistance) is particularly obvious for the approach and diffusion of macromolecules and structurally rigid components (such as asphaltenes) (its diffusion coefficient is lower than TX-100 itself); on the contrary, for small molecules and flexible saturated components, it is easier to bypass or penetrate this barrier, thus showing faster diffusion capacity (its diffusion coefficient is higher than TX-100). Therefore, TX-100 is interpreted to regulate component migration mainly through a steric-hindrance-related effect associated with molecular size and flexibility. The molecular characteristics of sophorolipid are its carboxylic acid head and sugar ring. This structure gives it significant polarity and the ability to form hydrogen bonds. Our data shows that it has a special interaction with the most polar heavy oil component—resins. In the sophorolipid system, the diffusion behavior of resins is very prominent (the diffusion is the fastest and the MSD curve slope is large), which is likely because the polar head group of sophorolipid and the oxygen-containing and nitrogen-containing groups in the resins form a strong hydrogen bond network. This interaction is not a simple obstacle but to a certain extent promotes or “anchors” the activation and early detachment of resins at the interface, thus changing its dynamic path in the interface competition. Rhamnolipid has both an anionic head group (carboxylate) and a double-chain hydrophobic tail. This structural combination brings unique functions: its hydrophobic chain tends to interact with the aromatic-ring structure, while the negatively charged head group introduces an electrostatic effect. The simulation results show that in the later stage of simulation, the aromatic diffusion behavior in the rhamnolipid system changed significantly (the later diffusion coefficient was surpassed by rhamnolipid). This suggests that rhamnolipid may insert into the π-system of aromatic molecules through its hydrophobic chain, while relying on the electrostatic repulsion of the head group to effectively drive the desorption of aromatic molecules from the interface region in the later stage, showing a “late-stage drive” effect. An important cross-system commonality is the strong adsorption universality of the C001 crystal surface. Regardless of which surfactant or heavy oil component is used, the C001 crystal surface always shows the strongest interfacial enrichment tendency. This indicates that the geometry and electronic structure of the crystal surface itself are the key factors determining the adsorption site, and surfactants fine-tune the competitive adsorption on this basis.

In summary, in the mineral-free simulation system, the separation and regulation of heavy oil components by surfactants is essentially the combined result of the specific interaction mode (spatial resistance, strong polar interaction, and electrostatic and hydrophobic synergy) determined by their molecular structure and the inherent properties of each component (molecular size, polarity, and aromaticity) during interfacial competition. This understanding provides a clear molecular-level basis for the rational selection or design of surfactants with corresponding functional groups for specific heavy oil compositions.

#### 3.2.8. Molecular-Level Insights from Simulation in Mineral Systems

First of all, the mineral surface, especially its exposed C001 crystal surface, as a universal strong adsorption site, provides a common competitive platform for all surfactants and heavy oil components. This leads to two key consequences: first, it consolidates the dominant position of the C001 crystal surface in interfacial distribution, making the interfacial behavior of surfactants more crystallographically dependent; second, it significantly prolongs the time required for all molecules (including surfactants themselves) to reach diffusion equilibrium, because part of the molecules will prioritize these low-energy sites and temporarily “stay” at the interface, thus slowing down the rate of migration to the bulk phase.

Secondly, the mineral surface has significant selectivity for the modulation of different surfactant functions. For TX-100, the spatial resistance effect generated by its long chain is complicated in the mineral environment. The mineral surface not only becomes a potential anchor point for its long chain but also competes with it for interfacial space, resulting in the diffusion hindrance effect of TX-100 on macromolecules such as asphaltenes being more obvious in the early stage, but it is also more prone to dynamic adsorption–desorption, thus showing faster apparent diffusion than most components on the macro-MSD. For sophorolipid, the presence of the mineral surface may produce additional interaction with its polar head group, which to a certain extent competes or cooperates with the hydrogen bond network between it and resins. The simulation results show that this competition makes the “anchoring” and accelerated diffusion effects of sophorolipid on resins more concentrated in time, and the action interval (such as the peak interval of the C001 crystal surface) is clearer. For rhamnolipid, the negatively charged mineral surface may produce electrostatic interaction with its anionic head group, which does not weaken its core function of driving the later desorption of aromatics. Instead, it may advance and strengthen its “late-stage drive” effect by changing the local electric field, which is manifested in the realization of the aromatic diffusion surpassing that of the components in a shorter time scale.

In summary, the role of the mineral surface can be summarized as the dual function of “platform and sieve”. As a “platform”, it provides a stable and universal competitive stage for the interfacial process through specific crystal surfaces such as C001 and enhances the crystallographic foundation of adsorption selectivity. As a “sieve”, it filters and regulates the kinetic process of different surfactant–component pairs through its physicochemical heterogeneity and non-uniformity: on the one hand, it increases the diffusion resistance as a whole and prolongs the time window of separation kinetics; on the other hand, it selectively amplifies the differentiated regulation ability of specific surfactants (such as rhamnolipid) based on electrostatic, polar and other mechanisms.

Therefore, in the real oil–solid–water three-phase separation system, the high efficiency and selectivity of surfactants is the result of the synergy between its inherent molecular function and the mineral-interface microenvironment. This understanding emphasizes that the design and process optimization of high-efficiency separation agents for complex systems such as oil sands must comprehensively consider the structural characteristics of the mineral surface as a core variable. By rationally matching the molecular structure of surfactants and the mineral-interface properties, accurate regulation from the molecular scale to macroscopic efficiency can be achieved.

### 3.3. Experimental Validation of Surfactant-Assisted Heavy Oil Separation

In order to further support the molecular dynamics simulation results and establish the link between macroscopic separation behavior and the microscopic interfacial regulation mechanism, this paper supplements the recovery rate of heavy oil, contact angle, C/H atomic ratio, SARA four-component composition, zeta potential, AFM oil–solid adhesion, surface activity, and adsorption kinetics tests of the agent on the surface of calcite. The above experimental results can provide an experimental basis for the regulatory differences in the oil sand separation process of different surfactants from multiple perspectives, such as macroscopic recovery efficiency, wettability regulation, interfacial electrical changes, oil–solid adhesion strength, and surfactant adsorption behavior, and form a correspondence with descriptors obtained from molecular simulations, such as RDF, concentration distribution, and apparent diffusion coefficient.

#### 3.3.1. Heavy Oil Recovery, Contact Angle, C/H Ratio and SARA Composition

**Figure 11:**
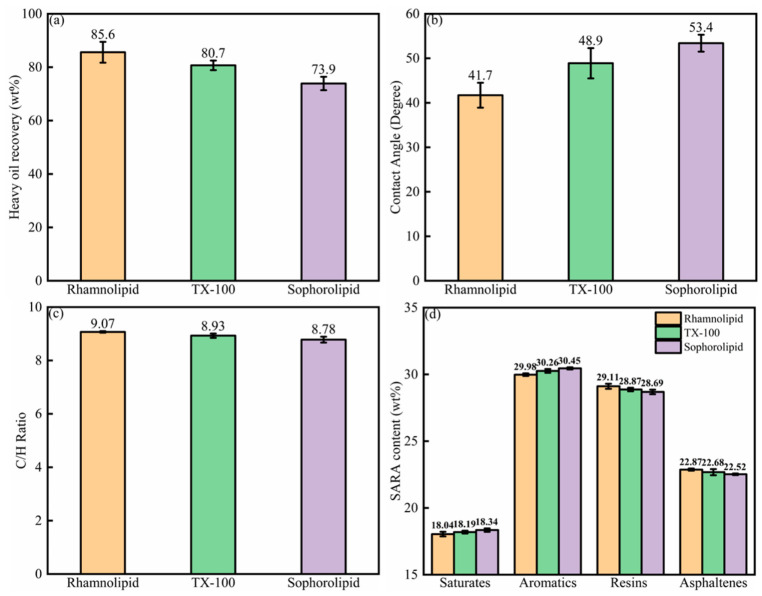
Experimental evaluation of heavy oil separation performance after surfactant treatment: (**a**) heavy oil recovery, (**b**) contact angle, (**c**) C/H ratio, and (**d**) SARA composition of recovered heavy oil.

There are obvious differences in the recovery rate of heavy oil after treatment with different surfactants. As shown in Figure 11a, after rhamnolipid treatment, the recovery rate of heavy oil reached 85.6 wt%, which is higher than the 80.7 wt% in the TX-100 system and 73.9 wt% in the sophorolipid system. The results show that rhamnolipid has a stronger promoting effect on oil-phase stripping and migration and can more effectively weaken the binding between heavy oil components and the mineral matrix, thus improving the efficiency of oil sand separation.

The contact angle test results further reflect the differences in the regulation of the wettability of the mineral surface by the three surfactants. As shown in Figure 11b, the contact angle of the rhamnolipid system is the lowest, which is 41.7°; the contact angles of the TX-100 system and the sophorolipid system are 48.9° and 53.4°, respectively. Usually, a lower contact angle indicates that the hydrophilicity of the mineral surface is enhanced, which is conducive to the spreading of the water phase on the solid surface and weakens the adhesion of the oil phase to the mineral surface. Therefore, the contact angle results are consistent with the results of the heavy oil recovery rate, indicating that the strong wettability regulation ability of rhamnolipid is one of the important reasons for improving the efficiency of heavy oil separation.

The results of the C/H atomic ratio also show certain system differences. As shown in Figure 11c, the C/H atomic ratio of the heavy oil obtained after treatment with rhamnolipid is 9.07, which is higher than the 8.93 in the TX-100 system and 8.78 in the sophorolipid system. A higher C/H atomic ratio usually reflects relatively high aromaticity or strong carbon enrichment characteristics in the oil phase. Therefore, the results show that rhamnolipid may be more conducive to promoting the migration and release of aromatic or heavier components. This experimental phenomenon coincides with the results of molecular dynamics simulations; that is, rhamnolipid can promote the interfacial migration of aromatic groups through the synergistic effect of hydrophobic action and electrostatic action.

The SARA four-component composition result is shown in Figure 11d. After treatment with the three surfactants, aromatics and resins in the recovered heavy oil are still the main components. In the rhamnolipid system, the saturate, aromatic, resin, and asphaltene contents are 18.04 wt%, 29.98 wt%, 29.11 wt%, and 22.87 wt%, respectively; in the TX-100 system, they are 18.19 wt%, 30.26 wt%, 28.87 wt%, and 22.68 wt%, respectively; and in the sophorolipid system, they are 18.34 wt%, 30.45 wt%, 28.69 wt%, and 22.52 wt%, respectively. Although the difference in the SARA composition among the three systems is relatively limited, the subtle changes in the contents of aromatics, resins, and asphaltenes still indicate that different surfactants may have a certain selective regulatory effect on the migration and release of different components of heavy oil.

Based on the results of the heavy oil recovery rate, contact angle, C/H atomic ratio, and SARA composition, it can be seen that rhamnolipid shows the best macroscopic separation performance among the three surfactants. Its high recovery rate of heavy oil, low contact angle, and high C/H atomic ratio together show that rhamnolipid can more effectively regulate the properties of the oil–water–solid three-phase interface and promote the migration and release of heavy oil components, especially aromatic components.

#### 3.3.2. Zeta Potential Analysis

To further reveal the regulatory effect of surfactants on the electrical environment of the oil–solid interface, this paper tests the zeta potential of asphaltene particles and mineral particles after treatment with different surfactants. The results show that all three surfactants can change the surface electrical state of oil-phase particles and mineral particles, indicating that they not only play a role by reducing interfacial adhesion or changing wettability but also affect the oil–solid interaction by adjusting the interfacial charge distribution.

For the asphaltene particle system, the zeta potential is −3.28 mV after treatment with rhamnolipid, while after TX-100 and sophorolipid treatment, the zeta potentials are 3.28 mV and 7.86 mV, respectively. The result shows that different surfactants regulate the surface electrical properties of oil-phase particles in different directions. After treatment with rhamnolipid, the asphaltene particles show negative charge, which may be related to the charge regulation effect of its anionic hydrophilic group on the oil-phase interface. This electrical change helps to enhance the electrostatic repulsion between oil-phase particles and between oil-phase particles and mineral surfaces, thus reducing the tendency of the oil phase to be readsorbed on the mineral surface.

For mineral particle systems, the mineral particles treated with all three surfactants show negative zeta potentials. Among them, the surface potential of mineral particles in the sophorolipid system is the lowest, which is −25.68 mV; those in the TX-100 and rhamnolipid systems are −17.46 mV and −11.28 mV, respectively. The enhancement of the negative charge of the mineral surface is usually conducive to improving the stability of its surface hydration layer and promoting the hydrophilicity of the mineral surface, thus weakening the adhesion of heavy oil components to the mineral surface.

The zeta potential results show that surfactants can regulate the electrostatic interaction at the oil–solid interface by changing the electrical state of oil-phase particles and mineral particles. This result provides experimental support for the explanation of the mechanism by which rhamnolipid promotes aromatic migration through hydrophobic–electrostatic synergy in molecular dynamics simulations.

#### 3.3.3. Oil–Solid Adhesion Force Measured by AFM

**Figure 12:**
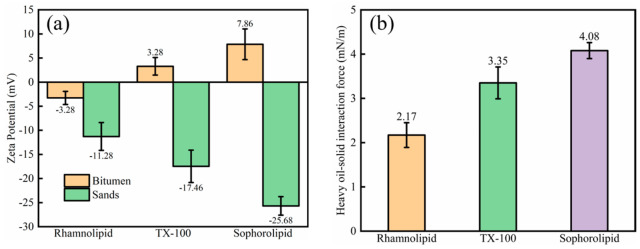
Interfacial electrostatic and mechanical characterization after surfactant treatment: (**a**) zeta potentials of bitumen/asphaltene particles and sand particles; (**b**) heavy oil–solid interaction force measured by AFM.

This paper uses atomic force microscopy to directly evaluate the influence of different surfactants on the adhesion strength of the oil–solid interface by testing the interaction force between the oil phase and the mineral surface. As shown in Figure 12, the oil–solid interaction force of the rhamnolipid system is the lowest, only 2.17 mN/m, which is significantly lower than the 3.35 mN/m in the TX-100 system and 4.08 mN/m in the sophorolipid system.

The lower oil–solid adhesion indicates that rhamnolipid can more effectively weaken the interfacial bonding strength between heavy oil components and mineral particles. This result is consistent with the heavy oil recovery rate and contact angle results; that is, lower oil–solid adhesion corresponds to higher heavy oil release efficiency and stronger interfacial stripping ability. The weak oil–solid force in the rhamnolipid system shows that it can significantly reduce the tendency of heavy oil to remain on the surface of minerals, thus promoting the detachment of the oil phase from the solid surface.

In contrast, although TX-100 can also reduce the oil–solid interfacial interaction to a certain extent, its effect is weaker than that of rhamnolipid. This may be related to the fact that TX-100 mainly relies on its non-ionic long-chain structure to produce steric hindrance and hydrophobic dispersion. The oil–solid interaction force of the sophorolipid system is the highest, indicating that its promoting effect on the separation of the overall oil–solid interface is relatively limited, but its polar functional groups may be more inclined to produce local regulation of specific polar components, such as resins.

The results of AFM adhesion provide direct macroscopic mechanical evidence for the molecular simulation results. In particular, the lowest oil–solid adhesion in the rhamnolipid system corresponds to its promoting effect on the migration of aromatic components and some heavier components at the interface in the simulation, which further shows that rhamnolipid can achieve more efficient heavy oil separation by weakening the oil–solid interfacial bonding.

#### 3.3.4. Surfactant Adsorption Kinetics on Calcite

**Figure 13:**
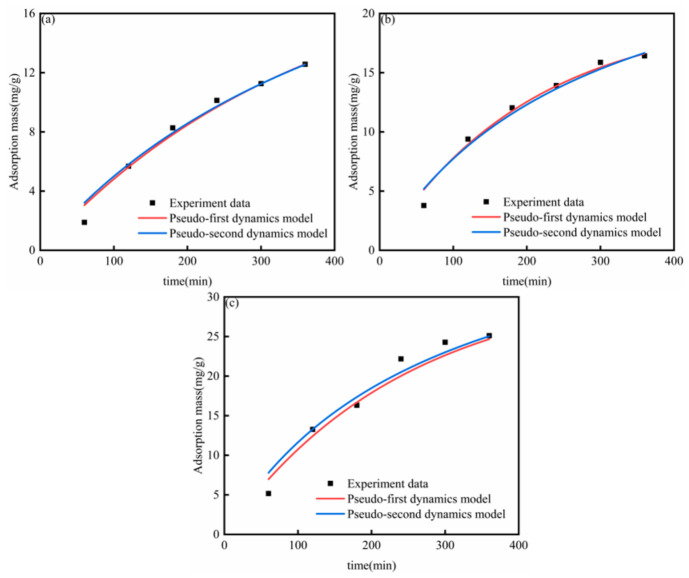
Adsorption kinetics of surfactants on calcite surfaces: (**a**) TX-100, (**b**) sophorolipid, and (**c**) rhamnolipid. The experimental data were fitted using pseudo-first-order and pseudo-second-order kinetic models.

This paper also examines the adsorption kinetic processes of three surfactants on the surface of calcite to further analyze the adsorption behavior of surfactants at the mineral interface. As shown in Figure 13, with the extension of the adsorption time, the adsorption amount of the three surfactants on the surface of calcite gradually increases and tends to stabilize in the later stage, indicating that their adsorption processes on the mineral surface have obvious time-dependent and gradual saturation characteristics.

The adsorption process is fitted using the pseudo-first-order kinetic model and the pseudo-second-order kinetic model. The results show that both models can describe the experimental adsorption data to a certain extent, but the pseudo-second-order kinetic model has a higher degree of agreement with the experimental data in the middle and late stages. This shows that the adsorption process of surfactants at the calcite interface is not only controlled by diffusion mass transfer but also may be jointly affected by factors such as the adsorption sites of surfactant molecules on the mineral surface, molecular orientation rearrangement, interfacial polarity, and electrostatic interactions.

The adsorption layer formed by the surfactant on the surface of the mineral can change the wettability, electrical state, and adhesion strength of the oil–solid interface on the calcite surface. Therefore, there is inherent consistency between the results of adsorption kinetics and the contact angle, zeta potential, and AFM adhesion results. Specifically, the gradual adsorption and interfacial rearrangement of surfactants on the mineral surface help to form a competitive adsorption layer, thus weakening the interaction between heavy oil components and calcite surfaces and promoting the migration of heavy oil components from the mineral interface to the solution phase.

In summary, the experimental results show that surfactant-assisted oil sand separation is a coupling process involving wettability transformation, interfacial electrical regulation, adsorption layer formation, and oil–solid adhesion reduction. Among the three surfactants, rhamnolipid shows the highest heavy oil recovery rate, the lowest contact angle, and the lowest oil–solid adhesion, indicating that it has stronger heavy oil stripping and interfacial migration promotion ability. The conclusion of this experiment is mutually confirmed with the results of molecular dynamics simulations, which further supports the mechanism by which rhamnolipid regulates the mineral interface through hydrophobic and electrostatic interactions, thus promoting the selective migration and debonding of heavy oil components.

#### 3.3.5. Correlation Between Experimental Results and Molecular Simulation Descriptors

This paper further addresses the problem of insufficient correlation between experimental characterization results and molecular simulation results by analyzing the macroscopic experimental results and the key descriptors obtained from molecular dynamics simulations. The experimental results mainly include the heavy oil recovery rate, contact angle, C/H atomic ratio, SARA composition, zeta potential, AFM oil–solid adhesion, and adsorption kinetic behavior; simulation descriptors mainly include the RDF peak strength, concentration distribution, interfacial enrichment behavior, and apparent diffusion coefficient. This correspondence does not mean that there is a strict one-to-one linear relationship between the experimental results and a single simulation parameter, but it is used to establish a semi-quantitative link between macroscopic separation behavior and the molecular-scale interfacial regulation mechanism.

As shown in Table 6, the rhamnolipid system has the highest heavy oil recovery rate, reaching 85.6 wt%, the lowest contact angle, 41.7°, and the lowest oil–solid adhesion, 2.17 mN/m. These experimental results show that rhamnolipid can more effectively promote the stripping of heavy oil from the mineral surface and enhance the migration ability of the oil phase in the aqueous phase. Correspondingly, the molecular simulation results show that the aromatic components and some heavy components in the rhamnolipid system have a more obvious interfacial migration tendency and strong apparent diffusion ability, indicating that its high macroscopic separation efficiency is closely related to component debonding and migration enhancement on the molecular scale.

The contact angle results reflect the ability of different surfactants to regulate the wettability of mineral surfaces. After treatment with rhamnolipid, the contact angle of the mineral surface is the lowest, indicating that it can more effectively enhance the hydrophilicity of the mineral surface, thus weakening the adhesion of the oil phase on the solid surface. The result coincides with the change in the interfacial concentration distribution in the simulation; that is, the enrichment and rearrangement of surfactant molecules at the mineral interface can change the oil–solid–water three-phase interface structure, thus promoting the migration of heavy oil components from the mineral surface to the solution phase.

Zeta potential results further reveal the regulatory effect of surfactants on the interfacial electrical environment. All three surfactants change the surface potential of oil-phase particles and mineral particles, indicating that the interfacial charge distribution plays an important role in the separation of heavy oil. In particular, rhamnolipid can affect the interaction between oil-phase particles and mineral surfaces by changing the local electrostatic environment because of its anionic hydrophilic group. The result is mutually confirmed with the mechanism proposed in the simulation to promote the migration of aromatic components through hydrophobic and electrostatic interactions.

The oil–solid adhesion measured by AFM can directly reflect the bonding strength between the heavy oil components and the mineral surface. The oil–solid adhesion of the rhamnolipid system is the lowest, indicating that it can significantly weaken the interfacial bonding between the oil phase and the mineral surface. The result is consistent with the strong component migration ability and interfacial debonding tendency observed in molecular simulations, indicating that the reduction of oil–solid adhesion is an important macroscopic performance to improve the efficiency of heavy oil separation.

The adsorption kinetics results show that the adsorption amount of the three surfactants on the surface of calcite gradually increases over time and finally tends to stabilize, and the pseudo-second-order kinetic model has better agreement with the experimental data in the middle and late stages. This shows that the adsorption of surfactants on mineral surfaces is not only controlled by diffusion mass transfer but also may be jointly affected by adsorption sites, molecular orientation rearrangement, and polarity or electrostatic interactions. The result is consistent with the enrichment behavior of surfactants at the mineral interface in the simulation, indicating that the formation of the adsorption layer helps to change the surface properties of the mineral and weakens the interfacial adhesion of heavy oil components.

In summary, there is a good correspondence between the experimental results and the molecular simulation descriptors. Macroscopically, rhamnolipid shows the highest heavy oil recovery rate, the lowest contact angle, and the lowest oil–solid adhesion; microscopically, it shows strong interfacial regulation ability, component migration ability, and hydrophobic–electrostatic synergy in simulations. Therefore, the experimental results and simulation results together show that rhamnolipid can promote the desorption and migration of heavy oil components from the mineral surface through multiple effects such as wetting change, electrostatic regulation, oil–solid adhesion reduction, and interfacial adsorption layer formation.

### 3.4. Integrated Discussion: From Molecular Mechanisms to Separation Selectivity

Based on the systematic study of non-mineral and mineral-containing systems, this study reveals the fundamental mechanism of three typical surfactants in the separation of heavy oil from the atomic scale. Beyond the characterization of macroscopic efficiency, our work has clarified a clear causal chain: the molecular structure of the surfactant determines its specific interaction mode with heavy-oil components, which regulates the competitive adsorption and diffusion kinetics of components at the interface, and finally manifests as observable separation-selectivity differences.

#### 3.4.1. Decoding the Separation Pathway: The Triad of Mechanisms

It should be emphasized that RDF and MSD analyses themselves cannot directly prove the existence of specific molecular interactions such as hydrogen bonding or electrostatic driving. Therefore, the relevant mechanisms discussed below should be understood as possible pathways based on evidence such as molecular structural characteristics, RDF results, concentration distribution, and apparent diffusion behavior, rather than deterministic mechanisms directly proved by a single descriptor. The expressions of spatial regulation, polarity/hydrogen-bond association, and electrostatic assistance used in this paper are mainly used to summarize the trend of molecular-scale changes presented by the simulation results and its reasonable mechanistic explanation.

It should be emphasized that RDF and MSD analyses themselves cannot alone be used as direct proof of the mechanism of action of specific molecules. For example, the RDF peak mainly reflects the local spatial correlation between molecules, while MSD mainly characterizes the molecular migration capacity and diffusion trend. The two cannot directly distinguish the relative contributions of hydrogen bonding, electrostatic interactions, or van der Waals interactions. Therefore, this paper describes mechanisms such as “steric hindrance screening”, “hydrogen-bond/polar-interaction-assisted debonding”, and “electrostatically assisted debonding” as possible mechanisms supported by multiple simulation descriptors, rather than deterministic mechanisms directly proven by a single RDF or MSD result.

For this reason, this paper further introduces supplementary simulation descriptors, including non-bonded interaction energy decomposition, hydrogen bond statistics, electrostatic descriptors, density redistribution at the calcite interface, adsorption energy attenuation, and steric accessibility parameters. See Tables S1–S4 for relevant results. The non-bonded interaction energy decomposition in Table S1 is used to distinguish the contributions of van der Waals interactions and electrostatic interactions to the interactions between different surfactants and SARA components; the hydrogen bond and electrostatic descriptors in Table S2 are used to evaluate the local polar effect and electrostatic response; the results of interfacial adsorption weakening and density redistribution in Table S3 are used to characterize the tendency of SARA components to migrate or desorb from the surface of calcite; and Table S4 further compares the component migration differences of different systems from the perspectives of diffusion capacity, steric accessibility, and steric hindrance.

Based on the comprehensive analysis of the above multiple descriptors, the role of TX-100 is more suitably expressed as a spatial regulation process dominated by van der Waals interactions and long-chain steric hindrance; the regulation of resin components by sophorolipid is more suitably expressed as an interfacial activation process assisted by polarity/hydrogen bond association; the regulation of aromatic components by rhamnolipid is more suitably expressed as an interfacial migration promotion process in which hydrophobic interactions and electrostatic interactions are jointly involved. Therefore, the three mechanisms of action proposed in this paper are not derived from RDF or MSD alone but are jointly supported by RDF, MSD, interaction energy decomposition, hydrogen bond/electrostatic descriptors, and interfacial density redistribution results.

This study establishes three dominant mechanisms that are completely different and can self-explain all observations. The core function of the non-ionic TX-100 is “spatial screening”. Its huge polyoxyethylene long chain forms a dynamic flexible barrier at the interface, which is extremely sensitive to molecular size and conformational rigidity. It has little hindrance to small molecules and flexible saturates and even provides a channel for rapid migration, while it produces significant diffusion blockage to large-volume and structurally rigid asphaltenes. This explains why in the TX-100 system, saturates always prioritize diffusion, and the separation of asphaltenes is the most difficult.

The mechanism of action of the biosurfactant sophorolipid can be summarized as “polar anchoring”. Its carboxylic acid head group and sugar-ring structure make it a strong polar unit, which can form a similar “anchoring” association with the most polar component in heavy oil resins through strong polar interactions such as hydrogen bonds. This effect is not simple adsorption but promotes the activation and early dissociation of resin molecules in the interfacial region, resulting in the fastest diffusion kinetics of resins in the sophorolipid system. This provides a unique molecular tool for the priority separation of the resin component.

Another biosurfactant, rhamnolipid, plays the role of “electrostatic driver”. Its molecules have both an anionic head group (carboxylate) and a double-chain hydrophobic structure. The hydrophobic chain tends to interact with the aromatic-ring structure, while the anionic head group introduces charge repulsion at the interface. This combination enables it to effectively insert into the π electron cloud region of the aromatic molecules and strongly drives the aromatic molecules to desorb from the interface through electrostatic repulsion in the later stage of simulation, which shows that its diffusion coefficient surpasses that of rhamnolipid itself in the later stage. This provides a new pathway for targeted driving of aromatics based on electrostatic regulation.

#### 3.4.2. The Universal Key: C001 Crystal Face Adsorption

A prominent law that runs through all simulation systems (with or without minerals, with any surfactant or component) is the adsorption universality of the C001 crystal surface. In all cases, the C001 crystal surface shows the strongest adsorption affinity for all molecules. This phenomenon is rooted in crystallography: the C001 crystal surface usually has the most stable and dense atomic arrangement and specific electron distribution, which provides it with the minimum surface energy and the most favorable molecular adsorption geometric site.

This discovery goes beyond the scope of specific surfactant research and has important universal guiding significance. It shows that in the development of solid materials (such as adsorbents, catalyst carriers or separation membranes) for oil sand separation or other solid–liquid separation processes, the active design and exposure of a high proportion of C001 crystal surfaces may be a universal and effective strategy, which can significantly enhance the material’s ability to capture interfacial-active molecules or target separation components, so as to fundamentally improve the separation efficiency.

#### 3.4.3. Implications for the Molecular Design of Next-Generation Surfactants

The “screening”, “anchoring” and “driving” mechanisms revealed by this research provide a clear blueprint for the rational design of highly selective and intelligent surfactants for complex heavy oil systems. The future design should no longer be limited to finding molecules with single efficacy but should be committed to integrating multi-functional fragments.

For example, it can be envisaged to rationally combine or transform the polar “anchoring” head group of sophorolipid, the electrostatic “drive” head group of rhamnolipid and the long-chain “screening” skeleton of TX-100 through molecular engineering, so as to create a new type of “intelligent” surfactant. This molecule may have a programmatic function: first, use the polar head group to quickly anchor and remove the resins, and then use the electrostatic effect to drive the aromatic desorption; at the same time, its long-chain skeleton continues to screen and hinder the asphaltenes, and finally realizes the sequential separation of the four SARA components in time or space.

In summary, this study not only decodes the micromechanism of surfactants promoting heavy oil separation at the molecular level, but more importantly, it establishes a theoretical framework from molecular structure design to interfacial-behavior regulation to separation-performance optimization. This framework will promote the transformation of heavy oil separation technology from traditional empirical trial-and-error to a new paradigm based on molecular simulation and rational design.

## 4. Conclusions

From the perspective of algorithm-assisted molecular simulation analysis, this paper systematically analyzes the migration and desorption behavior of SARA components in heavy oil regulated by three types of surfactants at the calcite interface. Unlike relying solely on experimental results or static configuration explanations, this paper further converts trajectory information generated by molecular dynamics simulations into multidimensional descriptors and identifies different patterns of action of surfactants through their correspondences. This approach enables complex molecular movement processes to be expressed as comparable, generalizable, and interpretable data structures, thereby improving the quantification and logical consistency of the analysis of heavy oil separation mechanisms. From the interdisciplinary perspective of computer science, the significance of this study lies in transforming microbial behaviors in complex interface systems into feature sets that algorithms can process. Indicators such as RDF, MSD, concentration distribution, adsorption energy, hydrogen bond statistics, and electrostatic contribution are not only used to explain molecular mechanisms but also serve as input variables for subsequent prediction model development. Based on these descriptors, surfactant performance evaluation models, SARA component migration ability prediction models, and interface desorption efficiency classification models can be further constructed. Therefore, this paper provides a preliminary basis for the algorithmic research approach of “molecular simulation—feature extraction—mechanism identification—molecular design,” and also lays the methodological foundation for the intelligent screening and targeted design of heavy oil separation materials.

Through systematic molecular dynamics simulation, this study suggests the commonality and personality of the microscopic effects of three types of surfactants: non-ionic (TX-100) and biological origin (locust sugar lipids, mouse plum sugar lipids) and heavy oil SARA components. Beyond the superficial description of macro separation efficiency, the core of this work is to clarify how the molecular structure of a surfactant “encodes” the separation path of components through differentiated interface behavior. The study found that separation selectivity is rooted in the coordination of “interface layer structure regulation” and “compont dynamic screening”. Although all three affect the system through weak long-range effects, their dominant interaction tendencies are different:

As a typical non-ionic surfactant, TX-100′s huge polyoxyethylene chain forms a dynamic “molecular brush” structure at the interface. This structure mainly produces a spatial resistance effect, which primarily hinders the diffusion and proximity of asphalt with large molecular volume and structural rigidity. At the same time, it provides a faster migration channel for the saturation of small molecules, suggesting a steric-regulation effect related to molecular size and flexibility. Huai sugar lipids and mouse plum sugar lipids show the unique behavior of biological surfactants. The carboxylic acid head group and sugar-ring structure of locust sugar lipid may enable it to form polar/hydrogen-bonding-associated interactions with the polar groups in resins, resulting in the early co-adsorption of the two at the interface. The anionic characteristics and double-stranded hydrophobic structure of rat plum glycolipids enable it to more effectively insert into the π electron cloud dense area of aromatic particles and may facilitate the later-stage migration/desorption of aromatic molecules through electrostatic and hydrophobic contributions. This explains the order in which the diffusion behavior of the two is opposite to that of TX-100 (colloid/asphalt diffusion is faster in biological surfactants).

More importantly, this study discovered the “C001 crystal surface adsorption universality” across different types of surfactants. Simulation shows that regardless of whether the surfactant head base is charged or not and what the hydrophobic chain structure is, the C001 crystal surface becomes the lowest energy adsorption template because of its stable atomic coordinating geometry. This common law points out that in the design of solid phase separation materials, regulating the exposure of the C001 crystal surface may be a universal enhanced separation strategy.

In summary, this study provides a molecular-level interpretation of the different regulation behaviors of the three surfactants: TX-100 plays the role of “space screener”, locust sugar lip acts as the “polar anchorer”, and mouse plum sugar lipid plays the function of “static driver”. Through weak interaction with different component specificities (spatial resistance, hydrogen bonding, and electrostatic) and the common preference for the C001 crystal surface, the cognitive upgrade of complex heavy oil systems from “rough extraction” to “pathic separation” has been realized. This not only deepens the understanding of the microscopic role of surfactants but also points out a clear direction of molecular engineering: by rationally integrating different functional fragments (such as introducing specific head bases and regulating chain length and rigidity), intelligent surfactants with “sieving”, “anchoring” and “driving” functions can be designed, so as to now implement the highly selective and sequential separation of heavy oil components.

## Figures and Tables

**Table 1:** Construction of a non-calcite system.

	Saturates	Aromatics	Resins	Asphaltenes
Molecule number	10	10	10	10
Molecule weight (g/mol)	282.5	634.92	713	713
System 1 molecule number	10 × 60	10 × 60	10 × 60	10 × 60
TX-100 cell parameters (nm)	3.18 × 3.18 × 3.18	3.36 × 3.36 × 3.36	3.38 × 3.38 × 3.38	3.35 × 3.35 × 3.35
Sophorolipid cell parameters (nm)	3.36 × 3.36 × 3.36	3.52 × 3.52 × 3.52	3.56 × 3.56 × 3.56	3.56 × 3.56 × 3.56
Rhamnolipid cell parameters (nm)	3.94 × 3.94 × 3.94	3.52 × 3.52 × 3.52	4.06 × 4.06 × 4.06	4.16 × 4.16 × 4.16

**Table 2:** Construction of the calcite system.

	Saturates	Aromatics	Resins	Asphaltenes
Molecule number	10	10	10	10
System 2 molecule number	10 × 60 × 1000	10 × 60 × 1000	10 × 60 × 1000	10 × 60 × 1000
TX-100 cell parameters (nm)	7.28 × 2.99 × 11.14	7.29 × 2.99 × 15.03	7.28 × 2.99 × 11.87	7.28 × 2.99 × 12.00
Sophorolipid cell parameters (nm)	7.28 × 2.99 × 11.26	7.28 × 2.99 × 11.99	7.28 × 2.99 × 12.37	7.28 × 2.99 × 12.26
Rhamnolipid cell parameters (nm)	7.28 × 2.99 × 13.46	7.28 × 2.99 × 14.19	7.28 × 2.99 × 14.68	7.28 × 2.99 × 14.32

**Table 3:** RDF peak values of each component in the non-mineral system.

Systems	First Peak Value (SARA)	Second Peak Value (SARA)	First Peak Value (Solutions)	Second Peak Value (Solutions)
Saturates–TX-100	1.11 (58.30)	2.17 (14.64)	1.11 (9.63)	1.39 (1.91)
Aromatics–TX-100	1.11 (33.77)	1.41 (15.53)	1.11 (11.55)	1.39 (2.61)
Resins–TX-100	1.11 (29.72)	2.17 (7.16)	1.11 (11.78)	1.39 (2.68)
Asphaltenes–TX-100	1.11 (28.78)	1.41 (15.07)	1.11 (11.43)	1.39 (2.62)
Saturates–Sophorolipid	1.11 (110.72)	2.17 (27.35)	1.11 (8.20)	2.15 (2.05)
Aromatics–Sophorolipid	1.11 (59.13)	1.41 (27.20)	1.11 (8.91)	2.15 (2.20)
Resins–Sophorolipid	1.11 (51.38)	2.17 (12.24)	1.11 (8.99)	2.15 (2.22)
Asphaltenes–Sophorolipid	1.11 (52.60)	1.41 (27.93)	1.11 (9.35)	2.15 (2.29)
Saturates–Rhamnolipid	1.11 (68.89)	2.17 (16.86)	1.11 (9.66)	2.15 (12.30)
Aromatics–Rhamnolipid	1.11 (39.15)	1.41 (17.88)	1.11 (11.08)	2.15 (2.65)
Resins–Rhamnolipid	1.11 (34.70)	2.17 (8.24)	1.11 (68.56)	2.15 (2.75)
Asphaltenes–Rhamnolipid	1.11 (34.47)	1.41 (17.95)	1.11 (11.52)	2.15 (2.73)

**Table 4:** Apparent molecular diffusion coefficients of SARA in TX-100, sophorolipid and rhamnolipid systems with non-minerals.

	Coefficients of Diffusion (D × 10^−9^ m^2^/s)		
System	TX-100	Sophorolipid	Rhamnolipid
Saturates	0.0131	0.0069	0.0931
Aromatics	0.0100	0.0046	0.0143
Resins	0.0067	0.0069	0.0114
Asphaltenes	0.0079	0.0088	0.0165

**Table 5:** Apparent molecular diffusion coefficients of SARA in TX-100, sophorolipid and rhamnolipid with minerals.

	Coefficients of Diffusion (D × 10^−9^ m^2^/s)		
System	TX-100	Sophorolipid	Rhamnolipid
Saturates	0.6129	0.2219	0.2235
Aromatics	0.0678	0.0141	0.0236
Resins	0.0209	0.0290	0.0192
Asphaltenes	0.0111	0.0052	0.0475

**Table 6:** Experimental–simulation correlation for surfactant-assisted heavy oil separation.

Experimental Descriptor	Main Result	Related Simulation Descriptor	Mechanistic Implication
Heavy oil recovery	Rhamnolipid highest, 85.6 wt%	Aromatic diffusion/interfacial concentration	Stronger desorption/migration
Contact angle	Rhamnolipid lowest, 41.7°	Interfacial redistribution	Stronger wettability alteration
Zeta potential	Surfactant-dependent charge shift	Electrostatic contribution/RDF	Interfacial electrostatic regulation
AFM adhesion force	Rhamnolipid lowest, 2.17 mN/m	Apparent diffusion/desorption tendency	Weaker oil–solid adhesion
Adsorption kinetics	Quasi-second-order better fitting	Surfactant accumulation at calcite	Adsorption layer formation

## Data Availability

The data can be made available on request.

## Supplementary Materials

The following supporting information can be downloaded at: https://www.mdpi.com/article/10.3390/ma19143032/s1.
