# Supplementary material for: Algorithm-Assisted Molecular Dynamics Simulations Revealed the Microscopic Mechanism by Which TX-100 and Biosurfactants Regulate the Separation of Heavy Oils from Solids
Source: Materials (Basel). 2026 Jul 14;19(14):3032. doi: 10.3390/ma19143032 (PMC13413449; doi:10.3390/ma19143032)
Supplement: Supplementary file 1 [file materials-19-03032-s001.zip › materials-4356700-supplementary.pdf]

Supplementary Information

Algorithm-Assisted Molecular Dynamics Simulations Revealed the Microscopic Mechanism by Which TX-100 and Biosurfactants Regulate the Separation of Heavy Oils from Solids

Yutong Yang [1], Yuping Wang [2,*], Wu Wen [3] and Jinze Du [4,*]

[1] School of Telecommunications Engineering, Xidian University, Xi'an 710071, China; yutongwang8@163.com
[2] School of Computer Science and Technology, Xidian University, Xi'an 710071, China
[3] Hainan Provincial Industrial Research Institute, Haikou 570203, China; wuwenwwwwstu@163.com
[4] Zhejiang Institute of Tianjin University, Ningbo 315000, China
* Correspondence: wangyupingw891@163.com (Y.W.); dujinzededjz11@163.com (J.D.)

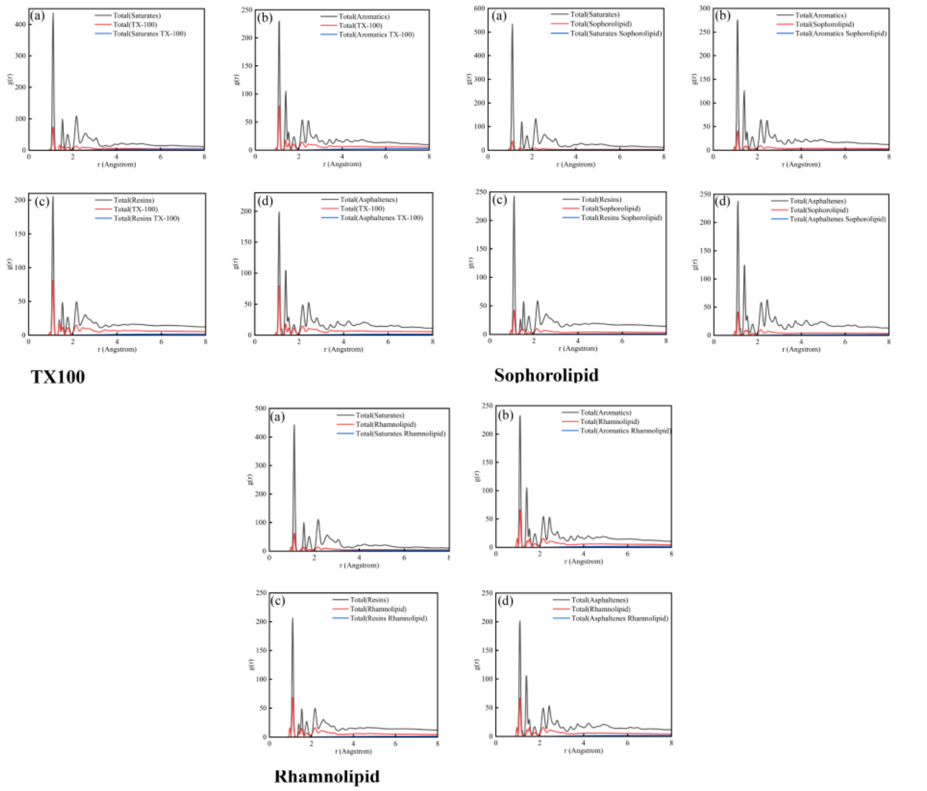

Figure S1. RDF between surfactants and SARA fractions in mineral systems: (a) saturates; (b) aromatics; (c) resins; (d) asphaltenes.

Academic Editor: Antonio Gil Bravo

Received: 19 May 2026
Revised: 22 June 2026
Accepted: 9 July 2026
Published: 14 July 2026

Copyright: © 2026 by the authors. Submitted for possible open access publication under the terms and conditions of the Creative Commons Attribution (CC BY) license.

Table S1. Non-bonded interaction energy decomposition between surfactants and SARA fractions in mineral-free systems.

Surfactant	SARA fraction	E_vdW (kcal/mol)	E_elec (kcal/mol)	E_total (kcal/mol)	vdW contribution (%)	Electrostatic contribution (%)	Dominant interaction interpretation
TX-100	Saturates	-25.38 ± 1.11	-1.72 ± 0.19	-27.10 ± 1.24	93.65	6.35	Weak vdW-dominated association with small flexible chains
TX-100	Aromatics	-39.64 ± 1.58	-3.05 ± 0.31	-42.69 ± 1.77	92.86	7.14	Aromatic association mainly controlled by dispersive interaction
TX-100	Resins	-35.91 ± 1.47	-5.47 ± 0.43	-41.38 ± 1.69	86.78	13.22	Weak polar participation superimposed on vdW association
TX-100	Asphaltenes	-53.26 ± 2.04	-4.29 ± 0.36	-57.55 ± 2.31	92.55	7.45	Strong vdW confinement and steric restriction
Sophorolipid	Saturates	-20.43 ± 0.92	-6.18 ± 0.44	-26.61 ± 1.08	76.78	23.22	Weak amphiphilic association
Sophorolipid	Aromatics	-33.52 ± 1.39	-10.34 ± 0.67	-43.86 ± 1.64	76.43	23.57	vdW-polar coupled association
Sophorolipid	Resins	-42.89 ± 1.83	-24.76 ± 1.16	-67.65 ± 2.42	63.40	36.60	Strong polar/H-bond-associated resin interaction
Sophorolipid	Asphaltenes	-39.78 ± 1.71	-15.63 ± 0.84	-55.41 ± 2.03	71.80	28.20	Moderate polar-vdW coupled association
Rhamnolipid	Saturates	-23.64 ± 1.06	-7.89 ± 0.53	-31.53 ± 1.26	74.98	25.02	Amphiphilic weak association
Rhamnolipid	Aromatics	-47.95 ± 2.07	-23.47 ± 1.09	-71.42 ± 2.68	67.14	32.86	Hydrophobic-electrostatic cooperative aromatic interaction
Rhamnolipid	Resins	-41.26 ± 1.74	-17.84 ± 0.91	-59.10 ± 2.15	69.81	30.19	Electrostatic-polar coupled interaction
Rhamnolipid	Asphaltenes	-44.81 ± 1.92	-20.93 ± 0.98	-65.74 ± 2.49	68.16	31.84	Electrostatic-vdW coupled interaction

Table S2. Hydrogen-bond and electrostatic descriptors for surfactant–SARA interactions.

Surfactant	SARA fraction	Average H-bond number	H-bond occupancy (%)	Average H-bond lifetime (ps)	Donor–acceptor distance (Å)	ESP complementarity index	Local electric-field response index	Mechanistic implication
TX-100	Saturates	0.03 ± 0.01	1.17	0.42	3.39	0.108	0.126	Hydrogen bonding and electrostatic effect are negligible
TX-100	Aromatics	0.14 ± 0.04	4.36	1.73	3.22	0.164	0.181	Weak local polar contact
TX-100	Resins	0.31 ± 0.06	9.58	2.69	3.09	0.217	0.245	Limited polar association
TX-100	Asphaltenes	0.22 ± 0.05	6.74	2.18	3.15	0.196	0.213	Steric/vdW effect remains dominant
Sophorolipid	Saturates	0.18 ± 0.03	5.23	2.04	3.17	0.279	0.284	Weak hydrogen-bond participation
Sophorolipid	Aromatics	0.53 ± 0.08	15.87	5.46	2.97	0.391	0.356	Moderate polar association

Surfactant	SARA fraction	Average H-bond number	H-bond occupancy (%)	Average H-bond lifetime (ps)	Donor–acceptor distance (Å)	ESP complementarity index	Local electric-field response index	Mechanistic implication
Sophorolipid	Resins	2.68 ± 0.24	66.49	21.37	2.74	0.718	0.639	Strong hydrogen-bond-assisted resin activation
Sophorolipid	Asphaltenes	1.26 ± 0.13	36.82	10.54	2.86	0.548	0.492	Partial polar anchoring
Rhamnolipid	Saturates	0.09 ± 0.02	2.41	1.28	3.28	0.337	0.372	Weak electrostatic response
Rhamnolipid	Aromatics	0.46 ± 0.07	13.29	5.12	3.01	0.783	0.741	Strong hydrophobic-electrostatic aromatic coupling
Rhamnolipid	Resins	1.38 ± 0.12	39.66	11.08	2.88	0.623	0.586	Electrostatic-polar coupled association
Rhamnolipid	Asphaltenes	1.17 ± 0.11	34.53	9.42	2.92	0.671	0.618	Electrostatic-vdW coupled retention weakening

Table S3. Calcite-interfacial adsorption weakening and density redistribution after surfactant addition.

Surfactant	SARA fraction	Adsorption energy attenuation ΔE_ads (kcal/mol)	Density peak shift away from calcite Δz (nm)	Residual interfacial density ratio (I/I_0)	Surfactant enrichment factor at interface	Estimated detachment probability (%)	Interfacial desorption interpretation
TX-100	Saturates	6.28 ± 0.43	0.17 ± 0.02	0.814	1.26	18.73	Slight outward migration of small flexible molecules
TX-100	Aromatics	7.06 ± 0.49	0.14 ± 0.02	0.873	1.31	13.95	Limited aromatic displacement
TX-100	Resins	8.29 ± 0.56	0.12 ± 0.01	0.902	1.34	11.24	Interfacial retention remains obvious
TX-100	Asphaltenes	4.71 ± 0.37	0.09 ± 0.01	0.937	1.38	6.52	Strong confinement near calcite is maintained
Sophorolipid	Saturates	8.17 ± 0.54	0.24 ± 0.02	0.758	1.47	23.41	Moderate displacement of weakly adsorbed saturates
Sophorolipid	Aromatics	13.68 ± 0.82	0.33 ± 0.03	0.684	1.56	27.82	Partial aromatic release
Sophorolipid	Resins	34.19 ± 1.74	0.61 ± 0.05	0.421	1.89	51.67	Preferential resin desorption from calcite interface
Sophorolipid	Asphaltenes	16.53 ± 0.93	0.29 ± 0.03	0.706	1.62	21.39	Partial weakening of asphaltene adhesion
Rhamnolipid	Saturates	10.94 ± 0.65	0.31 ± 0.03	0.693	1.71	29.58	Moderate outward migration

Surfactant	SARA fraction	Adsorption energy attenuation ΔE_ads (kcal/mol)	Density peak shift away from calcite Δz (nm)	Residual interfacial density ratio (I/I₀)	Surfactant enrichment factor at interface	Estimated detachment probability (%)	Interfacial desorption interpretation
Rhamnolipid	Aromatics	32.76 ± 1.61	0.64 ± 0.05	0.397	2.08	56.43	Strong aromatic displacement from calcite surface
Rhamnolipid	Resins	19.47 ± 1.04	0.35 ± 0.03	0.658	1.76	33.84	Moderate resin displacement
Rhamnolipid	Asphaltenes	25.18 ± 1.28	0.42 ± 0.04	0.604	1.82	38.27	Partial asphaltene detachment assisted by electrostatic interaction

Table S4. Diffusion, steric accessibility, and integrated mechanism-support descriptors.

Surfactant	SARA fraction	Apparent diffusion coefficient D_app ($\times 10^{-9}$ m^2/s)	Diffusion equilibrium time (ps)	Accessible free-volume ratio	Steric barrier index	Component mobility rank in each surfactant system	Mechanism-support score	Revised mechanistic description
TX-100	Saturates	1.372 ± 0.061	1286	0.736	0.183	1	0.781	Small saturates can pass through the TX-100 interfacial layer more easily
TX-100	Aromatics	1.046 ± 0.052	1457	0.618	0.341	2	0.692	Aromatics are moderately restricted by TX-100 molecular chains
TX-100	Resins	0.724 ± 0.039	1629	0.542	0.476	3	0.613	Resin migration is constrained by combined size and polarity
TX-100	Asphaltenes	0.612 ± 0.034	1784	0.459	0.592	4	0.844	Asphaltenes experience pronounced steric confinement
Sophorolipid	Saturates	0.983 ± 0.047	1322	0.694	0.217	2	0.574	Saturates are not the preferentially activated component
Sophorolipid	Aromatics	1.157 ± 0.055	1396	0.651	0.288	3	0.638	Aromatic mobility is moderately enhanced
Sophorolipid	Resins	1.846 ± 0.083	1171	0.583	0.356	1	0.912	Resins show preferential polar/H-bond-assisted activation
Sophorolipid	Asphaltenes	0.891 ± 0.044	1558	0.511	0.438	4	0.706	Asphaltene diffusion is only partially improved
Rhamnolipid	Saturates	1.094 ± 0.051	1247	0.706	0.201	3	0.621	Saturates undergo moderate amphiphilic regulation
Rhamnolipid	Aromatics	1.923 ± 0.087	1093	0.667	0.263	1	0.936	Aromatics show the strongest electrostatic-assisted displacement tendency

Surfactant	SARA fraction	Apparent diffusion coefficient D_app (×10⁻⁹ m²/s)	Diffusion equilibrium time (ps)	Accessible free-volume ratio	Steric barrier index	Component mobility rank in each surfactant system	Mechanism-support score	Revised mechanistic description
Rhamnolipid	Resins	1.286 ± 0.059	1384	0.609	0.329	2	0.748	Resins are moderately activated by polar-electrostatic coupling
Rhamnolipid	Asphaltenes	1.031 ± 0.048	1429	0.553	0.407	4	0.813	Asphaltenes show partial detachment but remain structurally restricted

Table S5. RDF peak values of each component in the mineral system.

Systems	First peak value (SARA)	Seconds peak value (SARA)	First peak value (solutions)	Seconds peak value (solutions)
Saturates-TX-100	1.11 (436.67)	2.17 (109.59)	1.11 (73.97)	1.39 (16.56)
Aromatics-TX-100	1.11 (230.10)	1.41 (104.92)	1.11 (11.48)	1.39 (17.73)
Resins-TX-100	1.11 (205.51)	2.17 (49.43)	1.11 (81.43)	1.39 (18.30)
Asphalteness-TX-100	1.11 (198.91)	1.41 (104.55)	1.11 (79.63)	1.39 (17.92)
Saturates-Sophorolipid	1.11 (534.00)	2.17 (133.71)	1.11 (39.30)	2.15 (9.49)
Aromatics-Sophorolipid	1.11 (276.03)	1.41 (126.15)	1.11 (41.40)	2.15 (10.02)
Resins-Sophorolipid	1.11 (242.74)	2.17 (58.98)	1.11 (42.50)	2.15 (10.23)
Asphalteness-Sophorolipid	1.11 (237.43)	1.41 (124.53)	1.11 (41.80)	2.15 (10.08)
Saturates-Rhamnolipid)	1.11 (442.70)	2.17 (110.88)	1.11 (62.61)	2.15 (14.76)
Aromatics-Rhamnolipid)	1.11 (232.69)	1.41 (105.51)	1.11 (66.53)	2.15 (15.76)
Resins-Rhamnolipid)	1.11 (206.63)	2.17 (49.90)	1.11 (68.56)	2.15 (16.14)
Asphalteness-Rhamnolipid)	1.11 (201.42)	1.41 (106.65)	1.11 (67.23)	2.15 (15.91)
